# A phosphinic acid bioisostere of hydroxyethylamine as a novel antiplasmodial scaffold: design, synthesis, and *in vitro* and *in silico* evaluation

**DOI:** 10.1039/d6ra09362k

**Published:** 2026-11-11

**Authors:** Moaz M. Abdou, Ahmed I. Khodair, Danwen Qiu, Rasha A. El-Saeed, Nawal Bahtati, Faez Iqbal Khan, Magdalini Matziari

**Affiliations:** a Egyptian Petroleum Research Institute Nasr City Cairo 11727 Egypt moaz.chem@gmail.com; b Chemistry Department, Faculty of Science, Kafrelsheikh University 33516 Kafrelsheikh Egypt; c Centre of Excellence for Molecular Innovation and Advanced Materials, Academy of Life and Natural Sciences, Xi'an Jiaotong-Liverpool University 111 Ren'ai Road, SIP Suzhou Jiangsu Province 215123 P. R. China; d Department of Chemistry, Faculty of Science, Mansoura University Mansoura 31556 Egypt; e Chemistry Department, Faculty of Science, Applied Science Private University Amman 11931 Jordan; f Centre of Excellence for Oncology and Immunology, Academy of Life and Natural Sciences, Xi'an Jiaotong-Liverpool University 111 Ren'ai Road, SIP Suzhou Jiangsu Province 215123 P. R. China Faez.Khan@xjtlu.edu.cn; g Department of Chemistry, Section of Organic Chemistry and Biochemistry, University of Ioannina 45110 Ioannina Greece matziari@uoi.gr

## Abstract

Malaria remains a major global health concern, and the emergence of drug-resistant *Plasmodium falciparum* strains highlights the need for new antimalarial scaffolds. In this study, a phosphinic acid-based analogue 2 was designed and synthesized through bioisosteric replacement of the hydroxyethylamine motif found in plasmepsin inhibitor scaffolds. The target compound was obtained through a multistep synthetic approach and evaluated for its antiplasmodial activity against the drug-resistant *P. falciparum* W2 strain. Compound 2 showed moderate whole-cell antiplasmodial activity with an IC_50_ value of 3.63 ± 0.11 µM. *In silico* ADMET analysis indicated favorable absorption-related properties and revealed several parameters requiring further experimental investigation. Molecular docking, 300 ns molecular dynamics simulations, and MM/PBSA calculations supported a plausible binding mode of compound 2 within the active site of plasmepsin II and provided insights into its interaction profile. Overall, this study supports the feasibility of phosphinic acid replacement as a bioisosteric modification strategy for plasmepsin inhibitor scaffolds. Compound 2 represents a preliminary lead scaffold for further optimization, where future studies including cytotoxicity evaluation, selectivity assessment, and enzymatic validation of plasmepsin II inhibition will be required.

## Abstract 2

Malaria remains a major global health concern, and the emergence of drug-resistant *Plasmodium falciparum* strains highlights the need for new antimalarial scaffolds.

## Graphical abstract

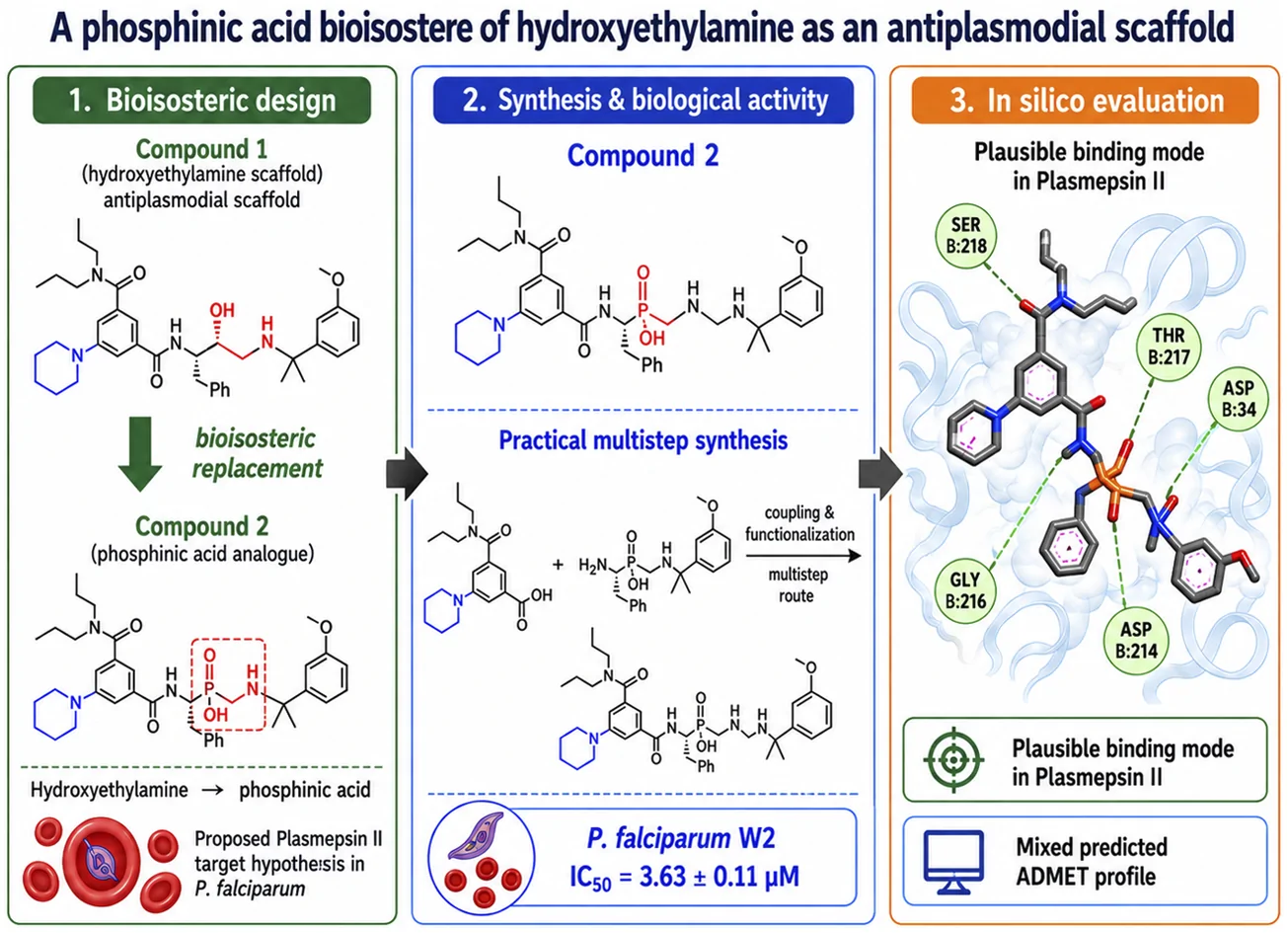

## 1. Introduction

Malaria remains one of the most serious global health challenges, particularly in tropical and subtropical regions, where it continues to affect millions of people annually.^1–5^ Despite substantial progress in malaria control, the continued emergence and spread of drug-resistant *Plasmodium falciparum* strains remains a major threat to effective case management.^6–10^ Although artemisinin-based combination therapies (ACTs) continue to represent the standard of care for uncomplicated malaria, the increasing concern over artemisinin partial resistance and reduced susceptibility to partner drugs highlights the urgent need for new antimalarial agents with improved pharmacological properties and resistance resilience.^11–14^

Among the chemical classes explored in antimalarial drug discovery, hydroxyethylamine-based compounds have attracted particular attention because of their inhibitory activity against plasmepsins, a family of aspartic proteases involved in parasite survival within infected erythrocytes.^15^ Representative compounds such as 1SR (TCMDC-134674) and compound 1 have provided valuable templates for structure-guided optimization.^16^ Nevertheless, despite their promise, hydroxyethylamine-containing scaffolds may still present limitations related to metabolic stability and overall developability, thereby restricting their broader therapeutic advancement.^17^

To address these limitations, increasing attention has been directed toward scaffold-modification strategies capable of preserving biological activity while improving physicochemical and pharmacokinetic properties. One such strategy is bioisosteric replacement, in which a problematic functional group is substituted with an alternative moiety that retains key molecular recognition features while potentially enhancing stability and drug-like behavior.^18^ In this context, phosphinic acids have emerged as attractive candidates because of their chemical stability and established value as enzyme-interacting motifs.^19–26^ Representative phosphinic acid-containing drugs and clinical candidates are shown in Fig. 1.

**Fig. 1:**
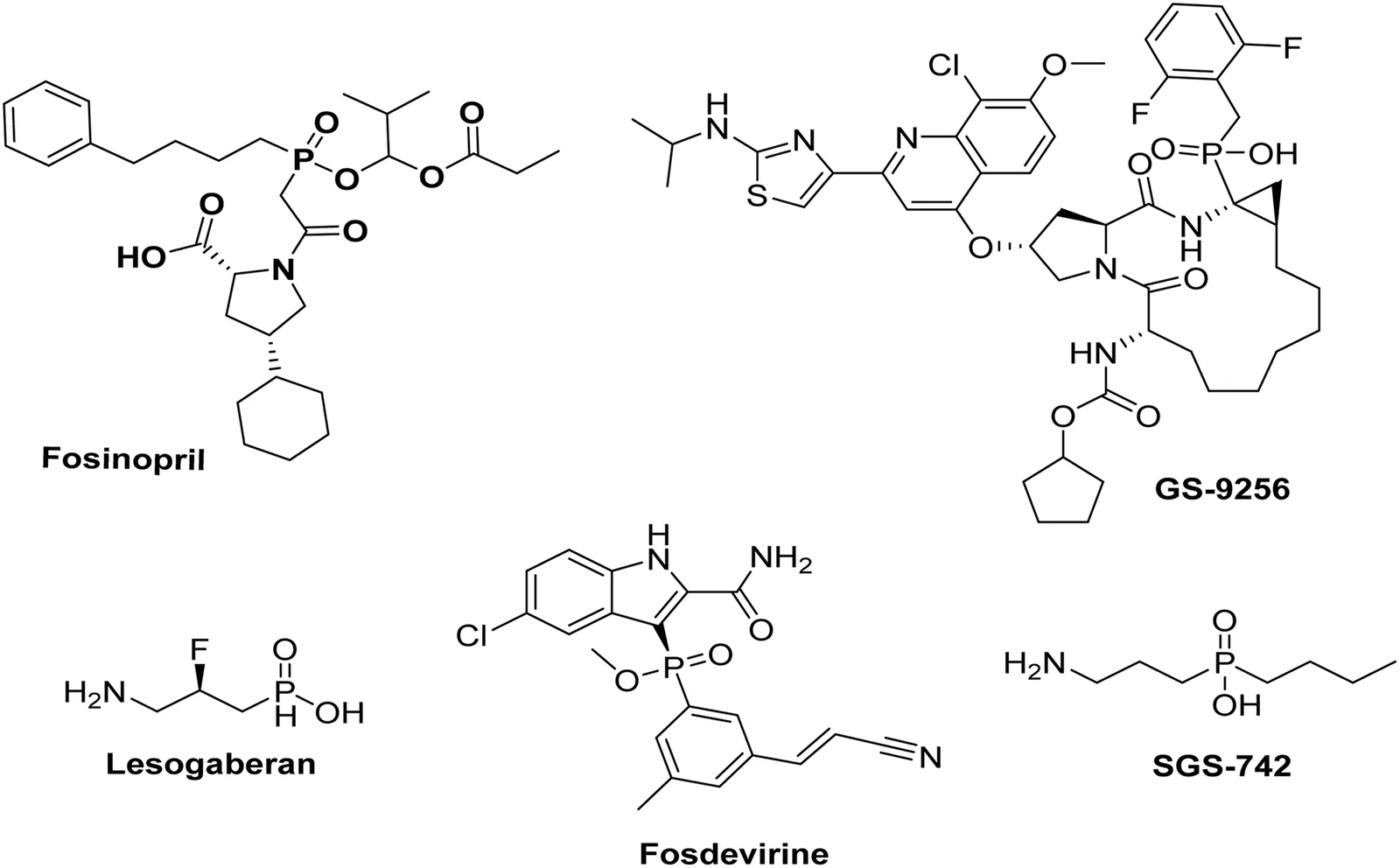
Representative phosphinic acid drugs currently found in clinical trials.

The rationale behind this bioisosteric replacement was to explore whether the phosphinic acid functionality could preserve key hydrogen-bonding interactions required for plasmepsin recognition while potentially improving physicochemical and metabolic properties.

### 1.1. Rational design of the phosphinic acid bioisostere

The hydroxyethylamine moiety is considered a key pharmacophoric element in plasmepsin inhibitor scaffolds because it mimics the tetrahedral intermediate formed during peptide bond hydrolysis and enables interactions with catalytic residues within the active site. Previous structural studies have demonstrated the importance of this central motif in maintaining effective binding interactions with plasmepsin enzymes.^15,16^

Based on these observations, we hypothesized that a C-amino phosphinic acid moiety could serve as a suitable bioisosteric replacement for the hydroxyethylamine functionality. The phosphinic acid group possesses a tetrahedral phosphorus center that can reproduce transition-state-like geometry, while the phosphoryl and hydroxyl functionalities provide additional hydrogen-bonding opportunities. Such characteristics may allow preservation of key enzyme recognition features while offering an alternative chemical framework for further optimization.

The hydroxyethylamine (HEA) motif is a well-established transition-state isostere in aspartic protease inhibitors. Its tetrahedral carbon center bearing a hydroxyl group mimics key structural features of the tetrahedral intermediate formed during peptide bond hydrolysis. Importantly, the hydroxyl group enables hydrogen-bonding interactions with the catalytic aspartate residues of aspartic proteases. In the case of plasmepsin II, crystallographic studies of the hydroxyethylamine-based inhibitor 1SR (PDB ID: 4CKU) have demonstrated interactions between the central hydroxyl group and the catalytic residues Asp34 and Asp214.^16^

In comparison, the C-amino phosphinic acid moiety possesses a tetrahedral phosphorus center containing phosphoryl (P=O) and hydroxyl (P–OH) functionalities, which provide an alternative structural arrangement capable of mimicking the tetrahedral geometry of the hydrolytic intermediate. Furthermore, phosphinic peptide analogues contain a hydrolytically stable P–C linkage, offering a non-cleavable alternative to the corresponding peptide bond. However, because P–O and P–C bonds are longer than the corresponding C–O and C–C bonds, the phosphinic acid moiety can be regarded as an expanded tetrahedral transition-state analogue rather than an exact geometrical replica. This structural feature has been discussed in the literature concerning phosphinic acid-based protease inhibitors.^27^ Moreover, crystallographic investigations of phosphorus-containing inhibitors bound to the aspartic protease penicillopepsin have provided experimental support for their ability to mimic tetrahedral transition-state interactions.^28^

From a physicochemical perspective, the phosphinic acid group is substantially more acidic and more readily ionized than the hydroxyl group of hydroxyethylamine. Consequently, the two functionalities differ in their electronic distribution, hydrogen-bonding behavior, polarity, and solvation characteristics. Depending on their protonation states, the phosphoryl and phosphinic acid oxygen atoms may participate in hydrogen-bonding interactions with catalytic residues. Nevertheless, these physicochemical differences may influence binding orientation, interaction stability, and molecular recognition within the enzyme active site.

Accordingly, compound 2 was designed by replacing the hydroxyethylamine motif of the previously reported plasmepsin inhibitor scaffold 1 with a C-amino phosphinic acid bioisostere (Fig. 2). This structural modification was intended to investigate whether phosphinic acid replacement could maintain antiplasmodial activity and provide a new starting point for medicinal chemistry optimization.

**Fig. 2:**
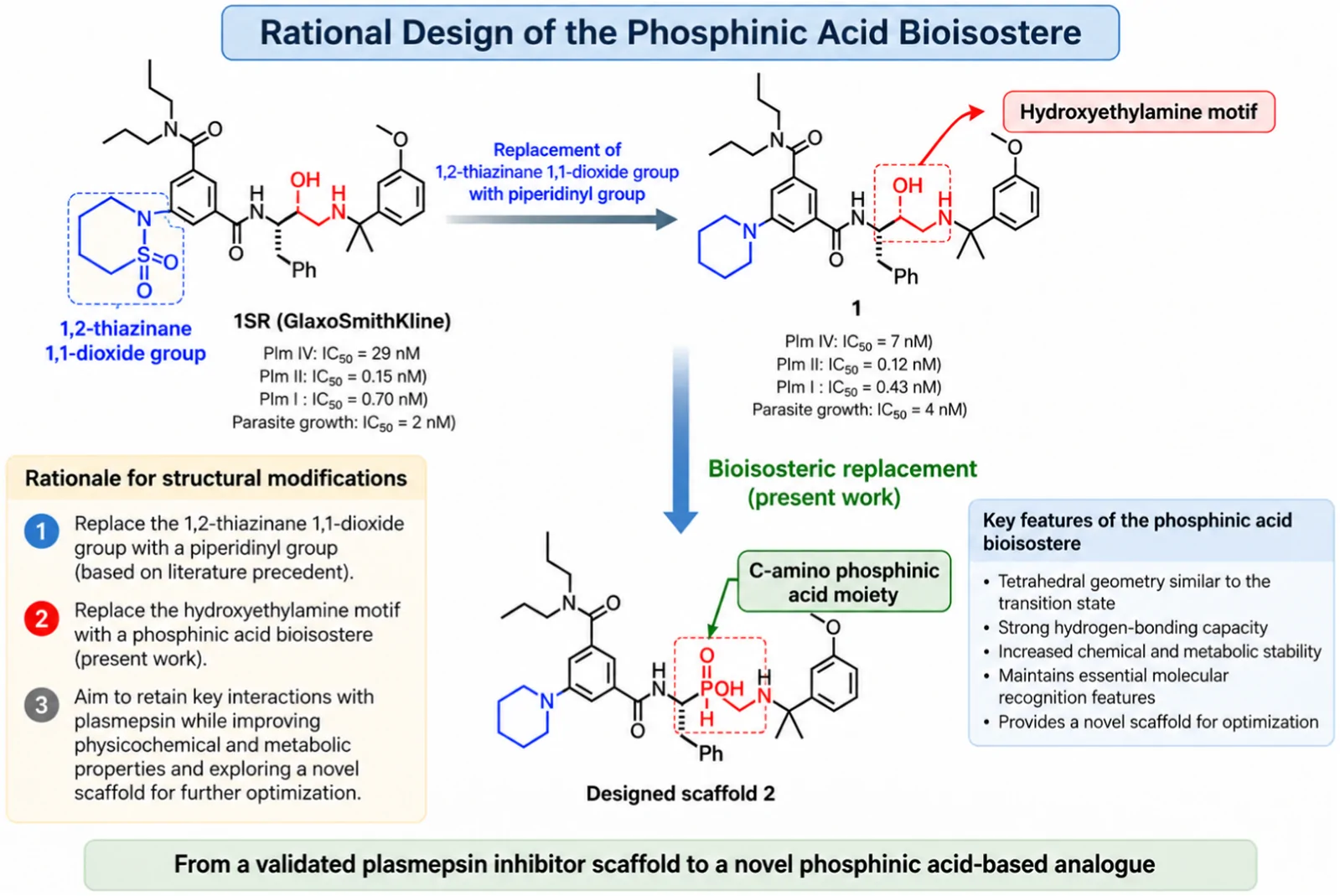
Structure-based rational design of compound 2. Starting from previously reported hydroxyethylamine-based plasmepsin inhibitor scaffolds, the hydroxyethylamine motif of compound 1 was replaced with a C-amino phosphinic acid moiety to evaluate phosphinic acid bioisosterism as a scaffold-modification strategy for antiplasmodial drug discovery.

In this study, we describe the design, synthesis, and biological evaluation of compound 2, a novel phosphinic acid-based analogue of hydroxyethylamine-containing plasmepsin inhibitors. The antiplasmodial activity of compound 2 was evaluated against the drug-resistant *P. falciparum* W2 strain. Furthermore, molecular docking, molecular dynamics simulations, MM/PBSA binding-energy calculations, and *in silico* ADMET prediction were performed to investigate its potential interaction profile and developability characteristics.

## 2. Results and discussion

### 2.1. Chemistry

The synthesis of the target compound 2 commenced with the preparation of methyl 3-(dipropylcarbamoyl)-5-(piperidin-1-yl)benzoate 7. At the outset, the preparation of 7 was planned according to a methodology previously reported (Scheme 1, route A).^16^ This approach consists of a Buchwald–Hartwig type amination reaction of dimethyl bromo-isophthalate 3 with piperidine (Pip.) to give 4 using palladium(ii) acetate and 2,2′-bis(diphenylphosphino)-1,1′-binaphthyl (BINAP) as a catalyst, and cesium carbonate as a base. Then, diester 4 can be monosaponified by the addition of one equivalent of sodium hydroxide to produce isophthalic monoester 5 which was transformed to amide 7 by amidation with dipropylamine in the presence of 1-ethyl-3-(3-dimethylaminopropyl)carbodiimide (EDC). The saponification step towards the synthesis of 5 proved to be problematic as it led also to the formation of diacid 6. The moderate yield towards the monoester 5 and the difficult purification steps made this route unsuitable for large-scale synthesis towards the isophthalic monoester 7.

**Scheme 1:**
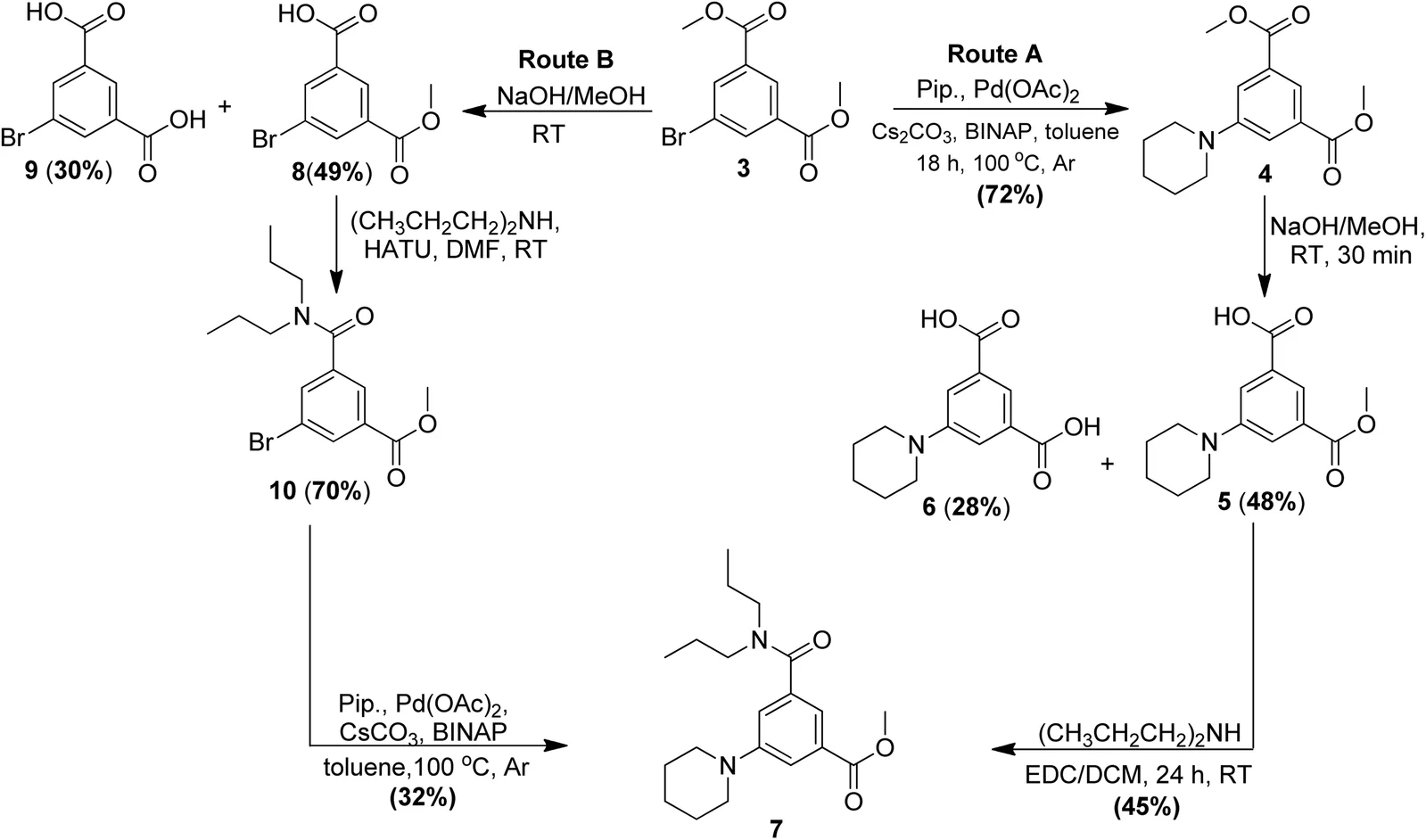
The two routes used for the synthesis of compound 7.

To minimize the formation of the undesired diacid 6 during saponification, we envisaged introducing the piperidinyl group after the saponification step (Scheme 1, route B). However, treatment of diester 3 with one equivalent of sodium hydroxide at room temperature (RT) afforded a mixture of the desired isophthalic monoester 8 and isophthalic acid 9.^29,30^ Following laborious purification by flash silica column chromatography (FSCC), compound 8 was isolated in 49% yield. Subsequent amidation of 8 with dipropylamine afforded amide 10 in 70% yield.^31^ The resulting intermediate 10 was then subjected to a Buchwald–Hartwig amination reaction with piperidine using palladium(ii) acetate and BINAP as the catalytic system and cesium carbonate as the base. However, this reaction furnished the desired compound 7 in only 32% yield, together with several unidentified by-products. Consequently, route B provided compound 7 in an overall calculated yield of approximately 11.0%. Although this approach enabled the synthesis of the desired intermediate, its low overall yield, by-product formation, and demanding chromatographic purification requirements limited its practical applicability for larger-scale preparation.

Hence, the imperative for a concise, cost-effective, and high-yielding route, featuring easily isolable and purifiable intermediates, is crucial for the successful synthesis of the target molecule. A reasonable choice for this was to start with the available isophthalic monoester 11 that would avoid the selectivity problems encountered before (Scheme 1, routes A and B).

A new synthetic strategy based on the “umpolung” concept is presented in Scheme 2 which was carried out starting from the reaction of the commercially available 5-nitro-isophthalic acid monomethyl ester 11 with oxalyl chloride at RT to an acid chloride intermediate which was treated *in situ* with dipropylamine in dichloromethane to generate the desired product 12 in excellent yield. The subsequent reduction step for the nitro group took place quantitatively using ammonium formate with palladium on activated carbon (Pd/C) in methanol to give the amine 13. *N*-Alkylation of the primary amine 13 to the tertiary amine 7 has been achieved by employing 1,5-dibromopentane in the presence of sodium dodecyl sulfate (SDS) and NaHCO_3_ in an aqueous medium. Saponification of the ester 7 produced the corresponding carboxylic acid 14. In contrast to the methods outlined above (Scheme 1, routes A and B), this highly efficient and scalable approach offers several advantages. It leverages easily accessible starting materials, operates under mild reaction conditions, eliminates the need for the transition metal Pd(OAc)_2_ and the extensive catalyst (BINAP), ensures a brief reaction time, achieves high yields, and facilitates a convenient work-up process.

**Scheme 2:**
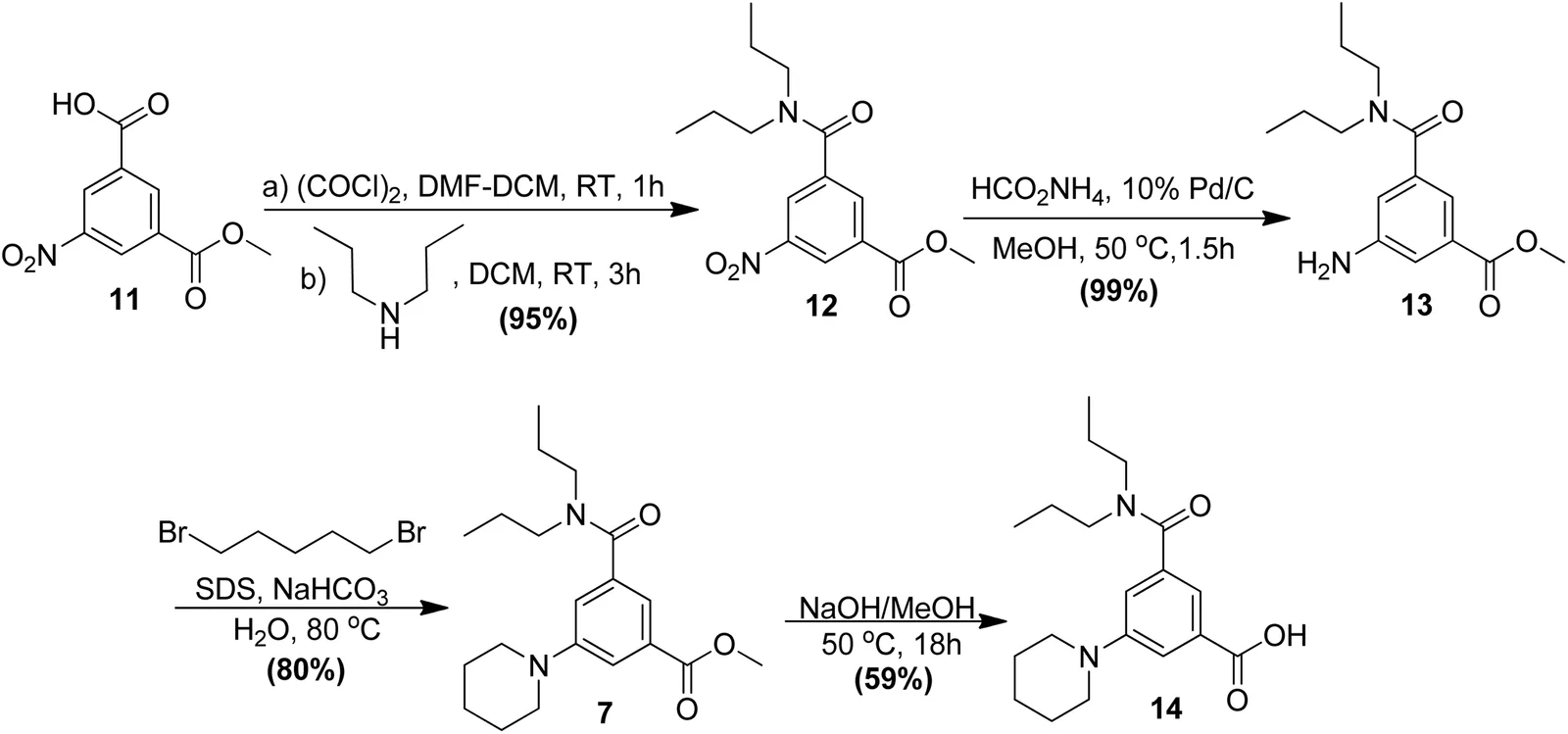
A new synthetic strategy for 5-piperidin-1-yl-*N*,*N*-dipropylisophthalamic acid 14.

To quantitatively assess the advantages of the optimized synthetic strategy, the overall yields, number of synthetic steps, and purification requirements of the three routes leading to intermediate 7 were compared, as summarized in Table 1.

**Table 1:** Quantitative comparison of the initial and optimized synthetic routes for the preparation of intermediate 7 a

Parameter	Initial route A	Initial route B	Optimized route
Synthetic sequence	3 → 4 → 5 → 7	3 → 8 → 10 → 7	11 → 12 → 13 → 7
Number of synthetic steps	3	3	3
Step 1 yield (%)	72	49	95
Step 2 yield (%)	48	70	99
Step 3 yield (%)	45	32	80
Overall yield (%)	15.6	11.0	75.2
Chromatographic purification steps	3	2	1
Purification requirements	Multiple column chromatography steps	Laborious chromatographic separation	One short silica-gel column
Relative yield improvement	—	—	4.8-Fold *vs.* A; 6.9-fold *vs.* B
Scale-up considerations	Limited by low yield and extensive purification	Limited by low yield and by-product formation	Improved laboratory-scale practicality; larger-scale validation required

a Overall yields were calculated by multiplying the reported individual step yields. The comparison concerns the preparation of intermediate 7, not the overall synthesis of final compound 2. The routes also begin from different starting materials.

A synthetic route toward the target phosphinic pseudotripeptide inhibitor 2 is shown in Scheme 3. The *tert*-carbinamine 16 was obtained through the reaction of methoxybenzonitrile 15 with cerium(iii)chloride and methyllithium in tetrahydrofuran.^16^ Mannich aminomethylation reaction of 16 with paraformaldehyde (PFA), and the optically pure (*R*)-Cbz *N*-protected aminophosphinic acid 17 in toluene was performed to give phosphinic acid 18 (Scheme 3). The Cbz group of 18 was removed by hydrogenolysis at RT in the presence of 10% Pd/C as a catalyst in methanol to furnish the free amino phosphinic acid 19 in 99% yield. HATU-mediated coupling of the phosphinic building block 19 with the acid 14 in the presence of *N*-ethylmaleimide (NEM) in DMF produced the target phosphinic acid 2. The (*R*)-configuration of compound 2 was assigned based on its synthesis from the optically pure (*R*)-configured aminophosphinic acid precursor 17. Although the subsequent transformations were designed to preserve the established stereogenic center, the enantiomeric purity of the final compound was not independently determined.

**Scheme 3:**
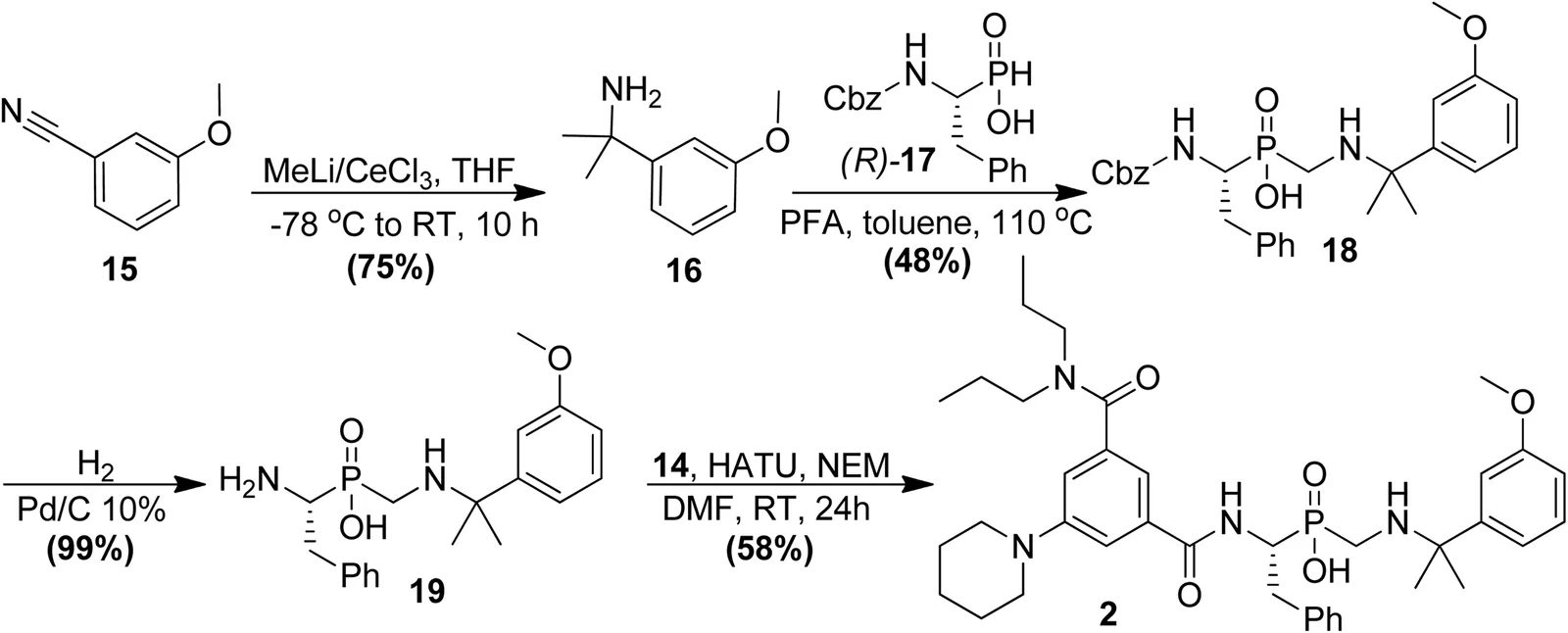
Synthesis of the target phosphinic pseudotripeptide inhibitor 2.

### 2.2. Biological evaluation

#### 2.2.1. Antiplasmodial activity

Compound 2 was systematically evaluated for its antiplasmodial activity against drug-resistant strains of *P. falciparum* (Table 2). Chloroquine (CQ) served as the standard reference, exhibiting an IC_50_ value of 0.141 ± 0.002 µM. The IC_50_ of compound 2 was determined by analyzing the logarithm of the dose/response relationship. Compound 2 inhibited the *P. falciparum* W2 strain with an IC_50_ of 3.63 ± 0.11 µM, indicating moderate whole-cell antiplasmodial activity under the assay conditions used.

**Table 2:** Antimalarial activity of compound 2 against *P. falciparum* IC_50_ is given ± the standard deviation

Compound	Structures	IC_50_ ± SD (µM)
2	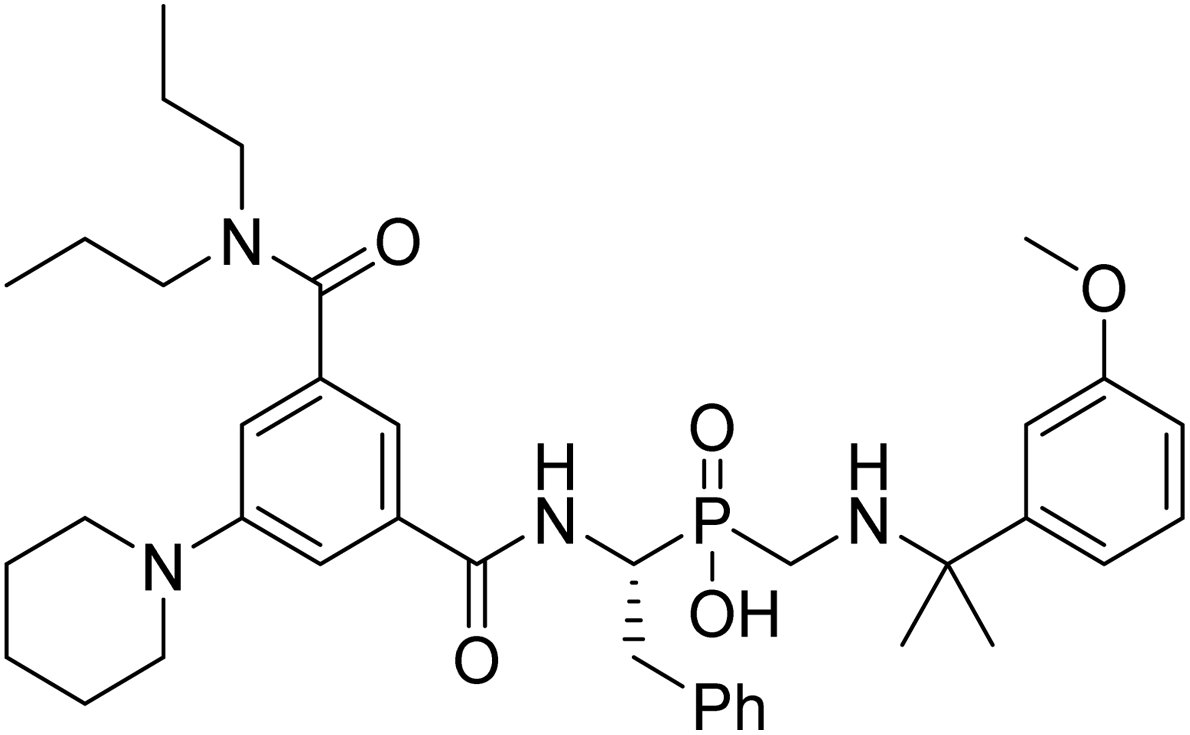	3.63 ± 0.11
Chloroquine	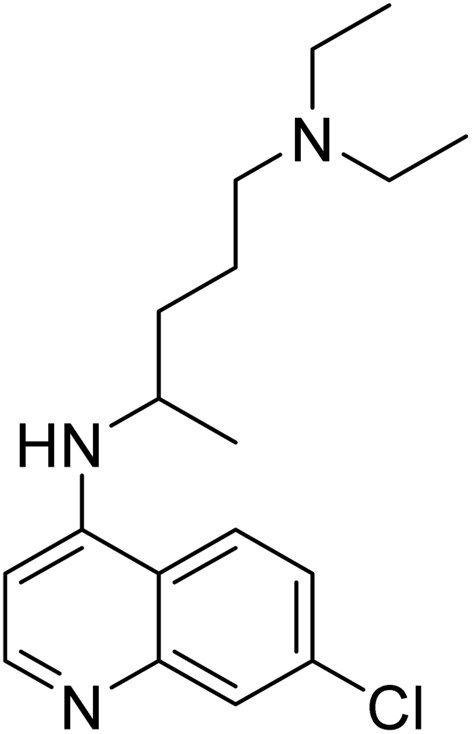	0.141 ± 0.002

Although compound 2 demonstrated measurable antiplasmodial activity, cytotoxicity evaluation against mammalian cell lines and subsequent selectivity index calculation were beyond the scope of the current preliminary study. These studies will be essential in future optimization campaigns to determine the therapeutic window and further assess the drug-development potential of this scaffold.

### 2.3. *In silico* prediction of parameters of ADMET

*In silico* prediction of pharmacokinetic and bioavailability parameters of 1 and 2 obtained from pkCSM are presented in Table 3. Generally, compound 1 and compound 2 exhibited comparable ADMET characteristics, including comparable intestinal absorption rates, shared substrate properties for P-glycoprotein and key CYP enzymes. They showed parallel distribution patterns with limited blood–brain barrier penetration and similar toxicity profiles, including hepatotoxicity risks but absence of AMES mutagenicity. Computational predictions indicated differences in several ADMET-related parameters between compounds 1 and 2; however, these findings require experimental validation. Additionally, 2 exhibited better CNS permeability and higher maximum tolerated dose, presenting reduced drug–drug interaction risks and improved pharmacokinetic properties for further development. The predicted differences in CNS permeability, CYP inhibition, and maximum tolerated dose should therefore be regarded as preliminary computational observations rather than evidence of improved pharmacokinetic performance.

**Table 3:** *In silico* prediction of ADMET parameters

	Compound 1	Compound 2
**Absorption**		
Water solubility, log mol L^−1^	−3.357	−3.269
Caco2 permeability (log *P*_app_ in 10^−6^ cm s^−1^)	0.668	0.91
Intestinal absorption, %	83.407	83.648
Skin permeability, log *K*_p_	−2.735	−2.735
P-glycoprotein substrate, yes/no	Yes	Yes
P-glycoprotein I inhibitor, yes/no	Yes	Yes
P-glycoprotein II inhibitor, yes/no	Yes	Yes

**Distribution**		
VDss, log L kg^−1^	1.217	1.116
Fraction unbound, *F*_u_	0.11	0.166
BBB permeability, log BB	−1.238	−1.364
CNS permeability, log PS	−2.592	−2.483

**Metabolism**		
CYP2D6 substrate, yes/no	Yes	Yes
CYP3A4 substrate, yes/no	Yes	Yes
CYP1A2 inhibitor, yes/no	No	No
CYP2C19 inhibitor, yes/no	Yes	No
CYP2C9 inhibitor, yes/no	Yes	No
CYP2D6 inhibitor, yes/no	No	No
CYP3A4 inhibitor, yes/no	Yes	Yes

**Excretion**		
Total clearance, log ml min^−1^ kg^−1^	1.187	0.709
Renal OCT2 substrate, yes/no	Yes	No

**Toxicity**		
AMES toxicity, yes/no	No	No
Maximum tolerated dose, log mg kg^−1^ day^−1^	−0.034	0.159
hERG I inhibitor, yes/no	No	No
hERG II inhibitor, yes/no	Yes	Yes
Oral rat acute toxicity, mol kg^−1^	2.09	2.014
Oral rat chronic toxicity, log mg per kg_bw per day	1.46	2.094
Hepatotoxicity, yes/no	Yes	Yes
Skin sensitization, yes/no	No	No
*T. pyriformis* toxicity, log µg l^−1^	0.285	0.287
Minnow toxicity, log mM	−0.465	−1.254

### 2.4. Molecular modelling and simulation

#### 2.4.1. Molecular docking analysis

Molecular docking computationally probes ligand–protein interactions, enabling drug candidate screening through binding affinity predictions to target proteins. Identification of functionally critical binding sites initiates protein functional analysis and provides the structural foundation for rational drug design.^32–37^ The 3D coordinates of the molecular target, plasmepsin II (PDB: 4CKU) was obtained from the Protein Data Bank. Molecular interactions of compound 1 and compound 2 with plasmepsin II were evaluated using the XP scoring function in Glide (Schrödinger Suite 2023.1). As a reference, compound 1 was docked into the same binding pocket with plasmepsin II. The Glide XP scoring algorithm yielded comparable docking scores for both compounds: −10.819 kcal mol^−1^ for 1 and −10.751 kcal mol^−1^ for 2. The docking scores and MMPBSA calculated binding free energies for the best pose for each compound are summarized in Table 4. The Molecular Mechanics/Poisson–Boltzmann Surface Area (MM/PBSA) binding free energy calculation revealed compound 1 possessed a more favorable Δ*G* (bind) value of −60.55 ± 4.85 kcal mol^−1^, compared to −40.77 ± 3.85 kcal mol^−1^ of compound 2. Docking results revealed that both 1 and 2 (Fig. 3) bind within the hydrophobic cavity of the plasmepsin II. Both 1 and 2 seem to conserve the characteristic hydrogen bonds with Asp34, Asp 214, Gly216 and Ser218, which are similar to 1SR. Moreover, compound 2 seems to form two additional hydrogen bonds with Gly36 and Thr217.

**Table 4:** The Glide XP docking scores and MM/PBSA binding free energies for top poses of 1 and 2 with plasmepsin II (PDB ID: 4CKU)

Target	Inhibitor	Glide XP docking score (kcal mol^−1^)	MM/PBSA binding free energies (kcal mol^−1^)
Plasmepsin II	1	−10.819	−60.55 ± 4.85
Plasmepsin II	2	−10.751	−40.77 ± 3.85

**Fig. 3:**
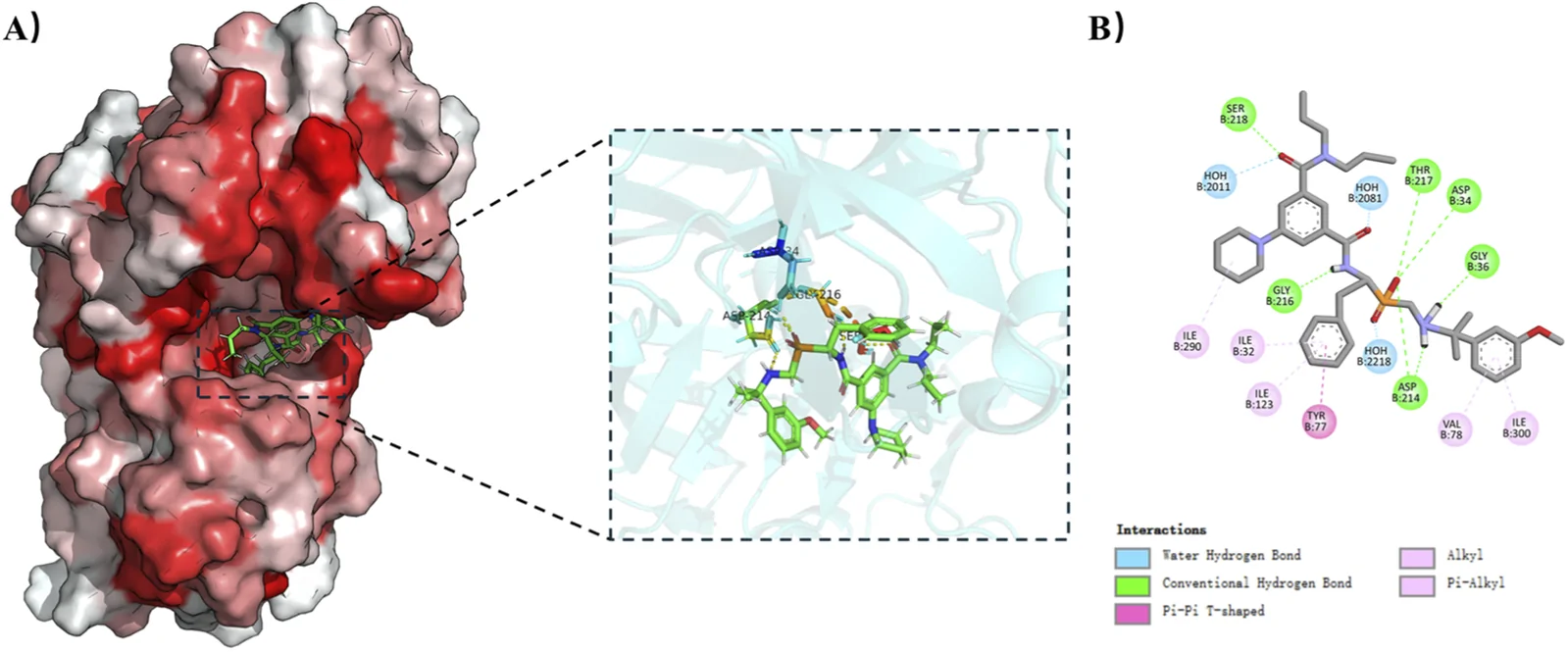
(A) Docked pose of compound 2 within the hydrophobic cavity of plasmepsin II, with color gradients indicating hydrophobicity: non-hydrophobic white to hydrophobic amino acids red. (B) Docked pose of compound 2 showing the molecular interactions with the active pocket of plasmepsin II (PDB ID: 4CKU).

#### 2.4.2. Global structural stability and conformation dynamics

Binding of ligands to the protein's active pocket may result in conformational changes. To explore the structural dynamics of plasmepsin II, plasmepsin II-compound 1, and plasmepsin II-compound 2, key structural and dynamic parameters (RMSD for protein backbone and ligands, *R*_g_, SASA, RMSF and hydrogen bonds) were monitored throughout the 300 ns molecular dynamics (MD) simulation at 37 °C. The RMSD of the protein backbone indicated enhanced stability upon ligand binding, with both ligand-bound complexes exhibiting lower average RMSD values compared to the unbound form (Fig. 4A). The mean RMSD values for plasmepsin II, plasmepsin II-compound 1, and plasmepsin II-compound 2 were estimated to be 0.225 nm, 0.204 nm, and 0.222 nm, respectively. In accord with the IC_50_ value difference *in vitro* between 1 and 2, the protein RMSD showed a more stable binding mode of 1 compared with 2. Similarly, the ligand RMSD of 1 (0.311 ± 0.030 nm) also suggested a more stable binding mode than 2 (0.360 ± 0.064 nm) (Fig. 4B). The *R*_g_ values revealed differences in compactness among the systems, the decrease of *R*_g_ in the unbound plasmepsin after 100 ns indicated structural contraction. The plasmepsin II-compound 2 exhibited higher *R*_g_ values compared to the plasmepsin II-compound 1 system, suggesting a less compact structure throughout the simulation (Fig. 4C). Minor differences were observed in SASA among the systems (Fig. 4D). In general, the global structural analysis demonstrated both the binding of 1 and 2 contributed to the stabilization of plasmepsin, while 2 showing a less pronounced effect and performance which is consistent with the *in vitro* results.

**Fig. 4:**
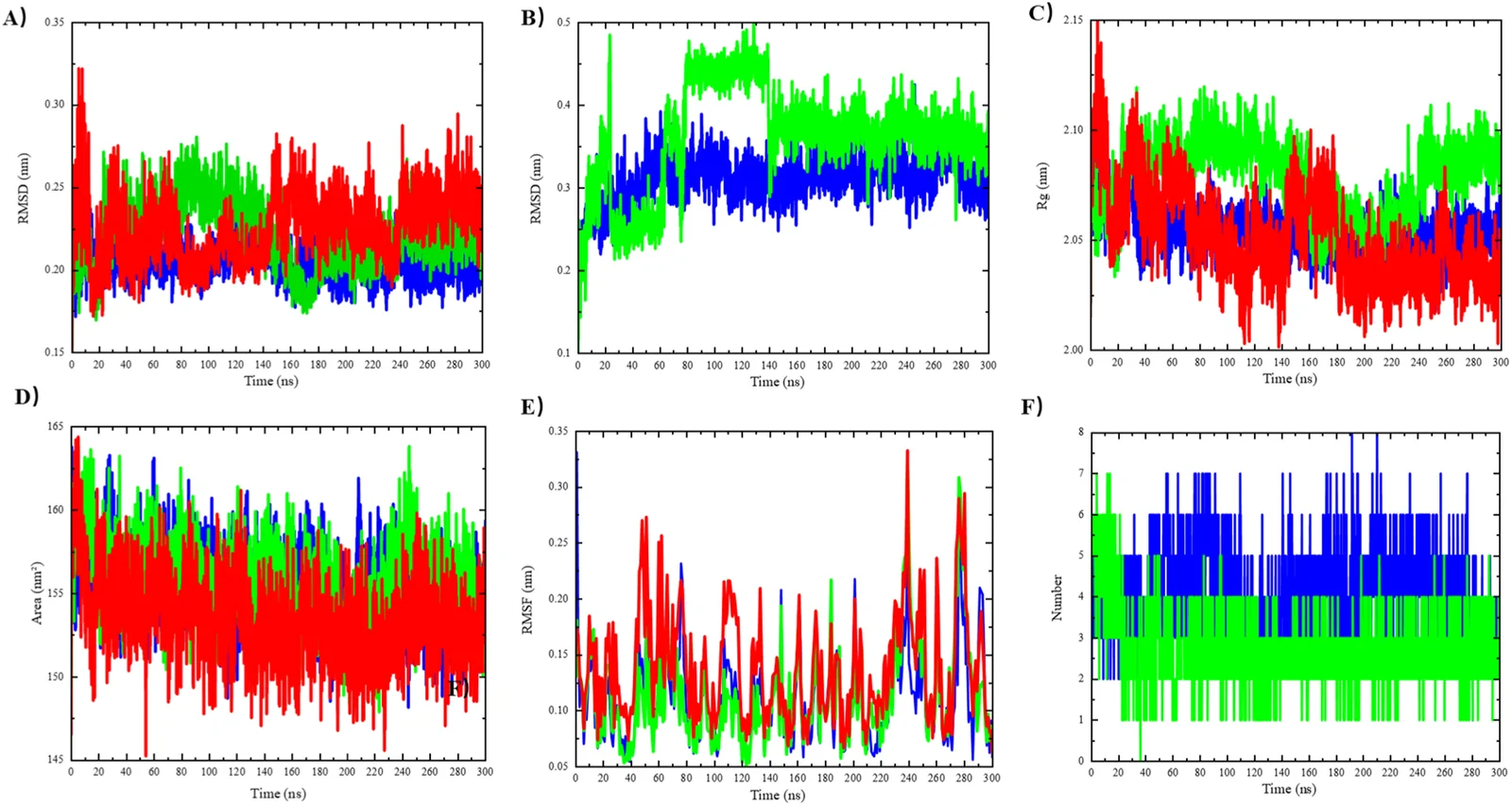
Structural dynamics of plasmepsin II. (A) RMSD plot for proteins *versus* time, (B) RMSD plot for ligands, (C) *R*_g_ plot *versus* time, (D) the solvent-accessible surface area (SASA) *versus* time, (E) RMSF *versus* residues, and (F) the hydrogen bonds estimation between ligands and protein. The values are calculated for the plasmepsin II (red), plasmepsin II-compound 1 (blue), and plasmepsin II-compound 2 (green), respectively.

The root-mean-square fluctuation (RMSF) values for the ligand–bound protein were calculated to reveal residue-level fluctuation during simulation. Binding of both compounds 1 and 2 led to a decrease in residue fluctuation at 107–115 aa, which may be attributed to stabilization of the ligand within the binding pocket (Fig. 4E). Hydrogen bonding critically contributes to the stability of ligand–bound protein conformations. Although docking predictions indicated the formation of 4 and 6 hydrogen bonds for 1 and 2, respectively. Hydrogen-bond analysis of the MD simulations showed the opposite trend, average hydrogen bonds count of 4.09 ± 1.03 and 2.80 ± 0.94 (Fig. 4F). This discrepancy suggests significant conformational dynamics instability in the binding of 2, a finding further supporting the MM/PBSA results, the global stability analysis, and the observed differences in *in vitro* activity.

#### 2.4.3. MM/PBSA

The binding affinities of compound 1 and compound 2 towards plasmepsin II were quantitatively evaluated using the MM/PBSA approach. The calculated binding free energies (Δ*G*) are summarized (Table 5). Consistent with *in vitro* efficacy, compound 1 exhibited a much higher binding affinity (−60.55 kcal mol^−1^) compared with the 2 (−40.77 kcal mol^−1^). Although both compounds exhibited comparable van der Waals interactions (−58.56 and −56.91 kcal mol^−1^), indicating similar shape complementarity and hydrophobic packing within the binding pocket, their binding affinities diverged significantly in terms of electrostatic contributions. Compound 1 demonstrated a profoundly favorable electrostatic energy (−102.78 kcal mol^−1^), suggesting the formation of critical polar interactions within the active pocket. In contrast, the near-neutral *E*_EEL_ value of 2 (−1.14 kcal mol^−1^) implies a notable lack of such electrostatic complementarity, indicating a distinct interaction mode of 2 toward plasmepsin II compared to 1.

**Table 5:** MM/PBSA-based binding free energy decomposition of 1 and 2 with plasmepsin II (PDB ID: 4CKU)

Compounds	Compound 1	Compound 2
*E*_VDWAALS_ (kcal mol^−1^)	−58.56 ± 1.99	−56.91 ± 1.04
*E*_EEL_ (kcal mol^−1^)	−102.78 ± 0.31	−1.14 ± 3.05
*E*_EPB_ (kcal mol^−1^)	98.38 ± 0.43	17.16 ± 0.53
*E*_ENPOLAR_ (kcal mol^−1^)	−9.16 ± 0.11	−9.52 ± 0.08
*G*_GAS_ (kcal mol^−1^)	−161.33 ± 2.01	−58.04 ± 3.32
*G*_SOLV_ (kcal mol^−1^)	89.23 ± 0.44	7.74 ± 0.53
Δ*H* (kcal mol^−1^)	−72.10 ± 2.06	−50.30 ± 3.27
Δ*G* (kcal mol^−1^)	−60.55 ± 4.85	−40.77 ± 3.85

#### 2.4.4. PCA of binding pocket dynamics

Principal component analysis (PCA) was employed to probe the differentiated collective dynamics of compound 1 and compound 2 toward plasmepsin II. The total eigenvalues (0.666 for compound 1*vs.* 0.461 for compound 2) indicated a significantly higher degree of overall conformational flexibility in the protein complex when bound to 1 (Table 6). The first principal component (PC1), which represents the most dominant collective motion, accounted for 37.2% of the total variance in compound 1 system. In contrast, PC1 for the 2 system explained only 22.8% of the variance, suggesting a less pronounced dominant motion and a more distributed mode of dynamics. Notably, the eigenvalue of the PC2 was higher for 2 (0.083) than for 1 (0.059), implying a relative shift in the hierarchy of collective motions between the two complexes. The cumulative variance explained by the first two PCs was also higher for 1 (46.0%) than for 2 (40.7%). The projection of the trajectories onto the first two principal components (PC1 and PC2) for 1 and 2 is also shown in Fig. 5, with the color gradient indicating simulation time (Table 6).

**Table 6:** Eigenvalues and cumulative explained variance of the top 5 principal components for the binding pocket dynamics

Principal component	Metric	Compound 1	Compound 2
PC1	Eigenvalue	0.247	0.105
	Cum. var. (%)	37.2	22.8
PC2	Eigenvalue	0.059	0.083
	Cum. var. (%)	46.0	40.7
PC3	Eigenvalue	0.050	0.046
	Cum. var. (%)	53.5	50.7
PC4	Eigenvalue	0.038	0.026
	Cum. var. (%)	59.2	56.3
PC5	Eigenvalue	0.035	0.020
	Cum. var. (%)	64.5	60.7
Total eigenvalues		0.666	0.461

**Fig. 5:**
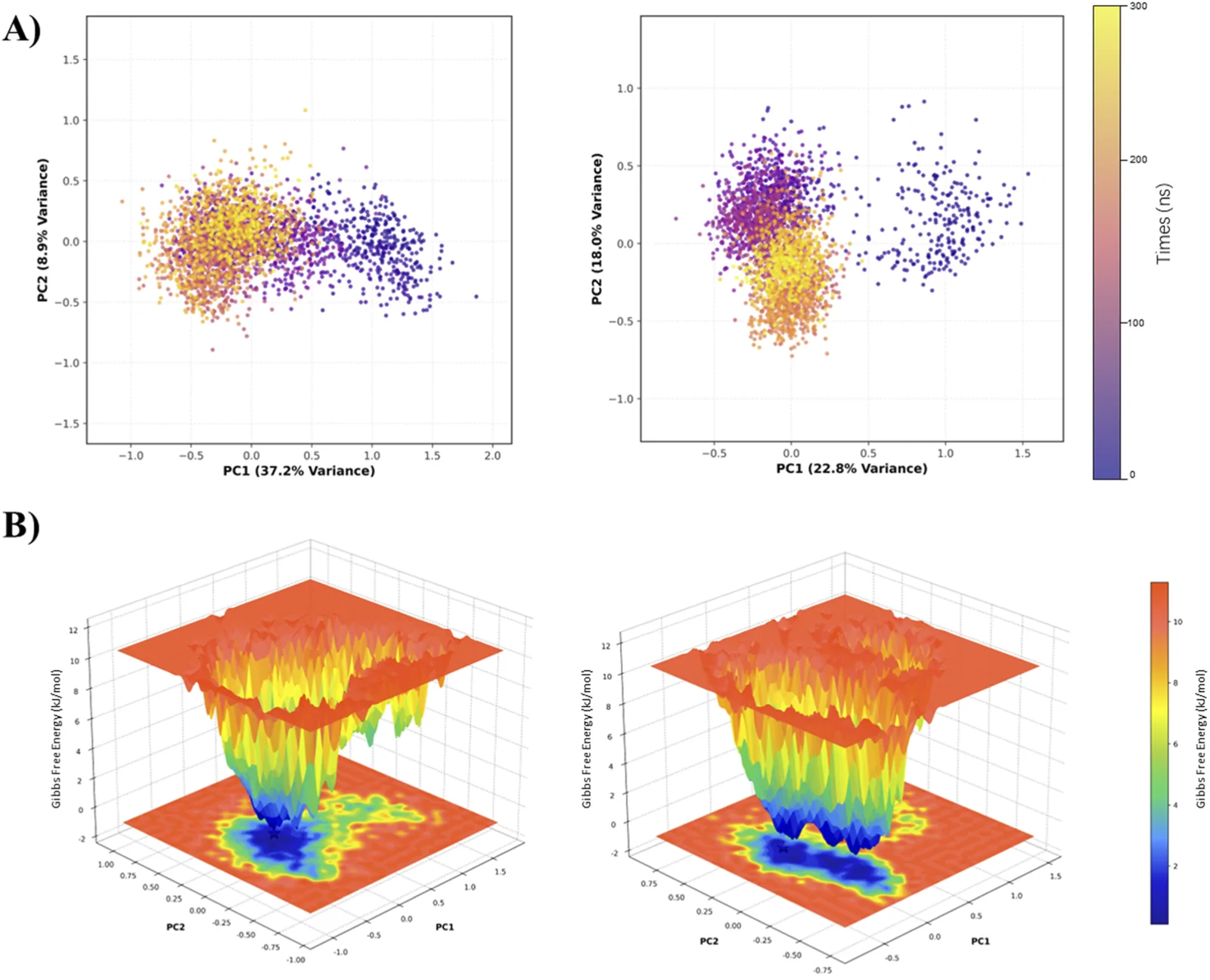
Principal component analysis of binding pocket: conformational space distribution (A) and Gibbs free energy landscape (B) during 300 ns simulation calculated for the plasmepsin II-compound 1 (left), and plasmepsin II-compound 2 (right), respectively.

The Gibbs free energy landscape (FEL) during 300 ns simulation was constructed to quantify the thermodynamic stability of the sampled conformations. Deep blue areas in the energy contour plot represent lower energy coordinates. The FEL for plasmepsin II-compound 2 featured a deep and stable global minimum, a hierarchical energy landscape is also observed with a metastable state located close proximity both in energy and conformational space. This may suggest a conformational selection mechanism in the binding of 2. The FEL for 1 was flatter with shallow global minimum (Fig. 5B). Together, the PCA and FEL results indicate the binding of 1 induces more concerted motions, while the binding of 2 results in a more restricted and complex dynamic landscape.

Although our molecular docking and MD simulations support plasmepsin II as a plausible target hypothesis for compound 2, it is important to acknowledge that our *in vitro* biological evaluation measured overall whole-cell antiplasmodial activity. Therefore, direct inhibition of plasmepsin II remains a proposed mechanism that requires experimental validation.

Although compound 2 exhibited promising antiplasmodial activity against the chloroquine-resistant *P. falciparum* W2 strain, its potential interaction with plasmepsin II remains a computationally supported hypothesis. The docking, molecular dynamics, and MM/PBSA results provide insights into possible molecular recognition within the enzyme active site but do not establish direct inhibitory activity or target engagement. Since a biochemical plasmepsin II inhibition assay was not performed, the observed whole-cell activity cannot be attributed exclusively to inhibition of this enzyme, and alternative molecular mechanisms cannot be excluded. Further biochemical and target-validation studies are therefore required to establish the mechanism of action of compound 2.

## 3. Experimental section

### 3.1. Synthesis and characterization

#### 3.1.1. Dimethyl 5-(piperidin-1-yl)isophthalate-piperidin-1-yl-isophthalic acid dimethyl ester (4)^16^

The ester 3 (1.99 g, 7.32 mmol), piperidine (0.73 ml, 7.32 mmol), and Cs_2_CO_3_ (3.58 g, 10.98 mmol) were placed into a dry round flak under argon. At the same time, Pd(OAc)_2_ (83.2 mg, 0.37 mmol), BINAP (228 mg, 0.37 mmol) were combined into another dry round flask and degassed under argon. Then dry toluene (5 ml) was added and the mixture was allowed to stir for 20 min under argon before being added to the first flask. The reaction mixture was heated at 110 °C for 24 h. The mixture was filtered, washed with AcOEt and the filtrate was concentrated under vacuum. The crude product was purified by FSCC (AcOEt/PE, v/v, 1/9) to afford 4 as a light yellow solid (1.46 g, 72%). ^1^H NMR (400 MHz, CDCl_3_) *δ* 1.60–1.65 (m, 2H), 1.68–1.83 (m, 4H), 3.28 (t, *J* = 5.30 Hz, 4H), 3.94 (s, 6H), 7.86 (s, 2H), 7.84 (s, 1H); ^13^C NMR (100 MHz, CDCl_3_) *δ* 24.11, 25.51, 50.33, 52.27, 120.64, 120.92, 131.16, 151.94, 167.11. HRMS (ESI/QTOF) *m*/*z*: [M + Na]^+^ calcd for C_15_H_19_NO_4_Na 300.1212; found 300.1188.

#### 3.1.2. 3-(Methoxycarbonyl)-5-(piperidin-1-yl)benzoic acid (5)^16^

Sodium hydroxide (291 mg, 7.26 mmol) was added on an ice bath to a suspension of 4 (2.01 g, 7.26 mmol) in MeOH (67.5 ml) and H_2_O (3 ml). The resulting suspension was allowed to stir at RT for 20 h. The methanol was removed *in vacuo* and the residual solution was partitioned between 10% KHSO_4_ (45 ml) and AcOEt (45 ml). Aqueous layer was washed twice with AcOEt (2 × 45 ml). The combined organic phase was washed with brine, dried over anhydrous Na_2_SO_4_ and evaporated. Purification by FSCC (EtOH/DCM, v/v, 5/95) gave the desired monomethyl isophthalate 5 as a white solid (920 mg, 48%). ^1^H-NMR (400 MHz, CDCl_3_) *δ* 1.59–1.69 (m, 2H), 1.69–1.80 (m, 4H), 3.31 (t, *J* = 5.41 Hz, 4H), 3.96 (s, 3H), 7.84 (s, 2H), 8.19 (s, 1H); ^13^C NMR (100 MHz, CDCl_3_) *δ* 24.12, 25.51, 50.00, 52.34, 121.23, 121.27, 121.68, 130.37, 131.31, 152.06, 166.79, 171.39. HRMS (ESI/QTOF) *m*/*z*: [M + K]^+^ calcd for C_14_H_17_NO_4_K 302.0795; found 302.0769.

#### 3.1.3. 5-(Piperidin-1-yl)isophthalic acid (6)

(By-product along with the synthesis of 5, white solid, 28%.) ^1^H NMR (400 MHz, DMSO-*d*_6_) *δ* 1.49–1.62 (m, 6H), 4.18 (t, *J* = 5.41 Hz, 4H), 7.65 (s, 2H), 7.87 (s, 1H), 13.11 (s, 2H); ^13^C NMR (100 MHz, DMSO-*d*_6_) *δ* 24.16, 25.42, 49.59, 120.08, 120.14, 132.39, 152.00, 167.52. HRMS (ESI/QTOF) *m*/*z*: [M + H]^+^ calcd for C_13_H_15_NO_4_H 250.1079; found 250.1068.

#### 3.1.4. 3-Bromo-5-(methoxycarbonyl)benzoic acid (8)^16^

To a solution of 3 (620 mg, 2.27 mmol) in THF–MeOH (1/1, v/v, 2 ml) was added dropwise 1 M NaOH_(aq.)_ (2 ml, 2.04 mmol). The mixture was allowed to stir at RT overnight. The volatiles were removed *in vacuo* and AcOEt was added and the mixture was extracted with H_2_O (2×). The combined aqueous phase was acidified using 2 M HCl (6 ml) to pH = 3, and extracted with AcOEt. The organic layer was dried over anhydrous Na_2_SO_4_ and the solvent was removed under reduced pressure. The residue obtained was purified by FSCC (DCM/MeOH, v/v, 50/1) to give 8 as a white solid (288 mg, 49%). M. p. 190–192 °C (lit. 191–193 °C). ^1^H NMR (400 MHz, DMSO-*d*_6_) *δ* 3.91 (s, 3H), 8.25–8.32 (m, 2H), 8.42 (s, 1H), 13.71 (s, H); ^13^C NMR (100 MHz, DMSO-*d*_6_) *δ* 53.38, 122.46, 129.06, 132.58, 136.51, 164.85, 165.58.

#### 3.1.5. 5-Bromoisophthalic acid (9)

(By-product along with the synthesis of 8, white solid, 30%), m. p. 278–280 °C. ^1^H NMR (400 MHz, DMSO-*d*_6_) *δ* 8.40 (s, 2H), 8.57 (t, *J* = 1.42 Hz, 1H), 13.76 (s, 2H); ^13^C NMR (100 MHz, DMSO-*d*_6_) *δ* 121.9, 128.7, 133.4, 135.6, 165.2.

#### 3.1.6. Methyl-3-bromo-5-[(dipropylamino)-carbonyl]benzoate (10)^16^

Dipropyl amine (1.1 ml, 7.72 mmol) and HATU (3.52 g, 9.27 mmol) were added at 0 °C to a solution of 9 (2.0 g, 7.72 mmol) in DMF (70 ml) and the resulting mixture was allowed to stir at RT overnight. The mixture was diluted with AcOEt, washed with H_2_O (3 times) and brine, dried over anhydrous Na_2_SO_4_, and concentrated under a vacuum to give 10 in 70% was used in the next step without purification. ^1^H NMR (400 MHz, CD_3_OD) *δ* 0.79 (t, *J* = 4.93 Hz, 3H), 1.03 (t, *J* = 4.93 Hz, 3H), 1.52–1.64 (m, 2H), 1.68–1.72 (m, 2H), 3.06–3.13 (m, 2H), 3.25–3.31 (m, 2H), 3.86 (s, 3H), 7.68–7.78 (m, 1H), 7.90–7.97 (m, 1H), 8.18–8.23 (m, 1H).

#### 3.1.7. Methyl 3-(dipropylcarbamoyl)-5-nitrobenzoate (12)

A suspension of 11 (1.35 g, 6.0 mmol) in DCM (50 ml) was treated with (COCI)_2_ (0.62 ml, 7.2 mmol) followed by a few drops of DMF. The reaction mixture was allowed to stir at RT for 1 h and then dipropylamine (2.79 ml, 20.4 mmol) was added and the resulting solution was allowed to stir for a further 30 min. The solution was then washed with 2 M HCI (65 ml), sat. NaHCO_3(aq.)_ (65 ml). The organic layer was dried over Na_2_SO_4_, filtered and concentrated under reduced pressure to afford the pure compound 12 as a pale yellow oil (1.74 g, 95%). ^1^H NMR (400 MHz, CDCl_3_) *δ* 0.80 (d, *J* = 6.11 Hz, 6H), 1.50 (d, *J* = 5.53 Hz, 4H), 3.09 (s, 2H), 3.42 (s, 2H), 3.92 (t, *J* = 6.44 Hz, 3H), 8.18–8.27 (m, 2H), 8.68–8.80 (m, 1H). ^13^C NMR (100 MHz, CDCl_3_) *δ* 10.88, 11.30, 20.58, 21.89, 46.70, 50.80, 52.83, 124.61, 125.46, 132.13, 133.08, 139.28, 148.11, 164.22, 167.90. HRMS (ESI/QTOF) *m*/*z*: [M + Na]^+^ calcd for C_15_H_20_N_2_O_5_Na 331.1270; found 331.1280.

#### 3.1.8. Methyl 3-amino-5-(dipropylcarbamoyl)benzoate (13)

Ester 12 (0.61 g, 2 mmol) was dissolved in 12 ml (EtOH/H_2_O, v/v, 2/1), NH_4_COOH (1.26 g, 20 mmol) was added. Palladium on carbon 10% (100 mg) was added to the resulting mixture and heated at 50 °C for 90 min. The reaction mixture was cooled to RT, and filtered through a pad of Celite. The solvent was removed under vacuum and the residue was dissolved in AcOEt (80 ml) and the resulting solution was washed with sat. NaHCO_3(aq.)_ (40 ml), dried over Na_2_SO_4_ and concentrated *in vacuo* to the pure compound 13 as a white solid (0.55 g, 99%). ^1^H NMR (400 MHz, CDCl_3_) *δ* 0.68 (t, *J* = 6.42 Hz, 3H), 0.80 (t, *J* = 6.42 Hz, 3H), 1.46 (d, *J* = 5.52 Hz, 2H), 1.60 (d, *J* = 5.52 Hz, 2H), 3.10 (s, 2H), 3.37 (s, 2H), 3.81 (s, 3H), 4.12 (s, 2H), 6.76 (s, 1H), 7.26 (s, 2H). ^13^C NMR (100 MHz, CDCl_3_) *δ* 10.97, 11.38, 20.63, 21.83, 46.25, 50.61, 52.06, 116.10, 116.71, 117.01, 131.01, 138.42, 147.37, 166.72, 171.18. HRMS (ESI/QTOF) *m*/*z*: [M + Na]^+^ calcd for C_15_H_22_N_2_O_3_Na 301.1528; found 301.1510.

#### 3.1.9. Methyl 3-(dipropylcarbamoyl)-5-(piperidin-1-yl)benzoate (7)

##### 3.1.9.1. Method 1 (ref. 16)

A mixture of 5 (497 mg, 1.89 mmol) and EDC (436 mg, 2.27 mmol) in DCM (15 ml) was allowed to stir at RT for 10 min then dipropylamine (229 mg, 2.27 mmol) was added and the resulting mixture allowed to stir at ambient temperature for 24 h. The reaction mixture was evaporated under reduced pressure. The obtained residue was purified by FSCC eluting with (AcOEt/DCM, v/v, 1/20). Product 7 was isolated as a colorless solid (229 mg, 45%).

##### 3.1.9.2. Method 2

In a dry flask under Ar, the 5-bromo-isophthalic acid dimethyl ester 10 (250 mg, 0.73 mmol), piperidine (0.073 ml, 0.73 mmol), and Cs_2_CO_3_ (360 mg, 1.10 mmol) were placed into a dry round flak under argon. At the same time, Pd(OAc)_2_ (8.32 mg, 0.037 mmol; 5 mol%), BINAP (2.28 mg, 0.037 mmol) were combined into another dry round flask and degassed under argon. Then dry toluene (0.5 ml) was added and the mixture was allowed to stir for 20 minutes under argon before being added to the first flask. The reaction mixture was heated at 110 °C for 24 h. The mixture was filtered, and washed with AcOEt and the filtrate was concentrated under a vacuum. The crude product was purified by FSCC using the eluent mixture (AcOEt/DCM, v/v, 1/20) to afford 7 as a colorless solid (81 mg, 32%).

##### 3.1.9.3. Method 3

The methyl ester 13 (0.69 g, 2.5 mmol) and NaHCO_3_ (0.25 g, 3 mmol) were taken up in H_2_O (7 ml). To this solution was added SDS (10 mg, 0.035 mmol) followed by 1,5-dibromopentane (1.36 ml, 5 mmol) and heated at 80 °C for 5 h. The reaction mixture was cooled and extracted with ethyl acetate. The crude reaction mixture was purified by passing it through a short column of silica gel and eluting with a mixture of (AcOEt/hexane, v/v, 1/20) to afford 7 (0.69 g, 80%) as a colorless solid. ^1^H-NMR (400 MHz, CDCl_3_) *δ* 0.70–0.80 (m, 3H), 0.91–1.01 (m, 3H), 1.41–1.53 (m, 2H), 1.54–1.60 (m, 2H), 1.61–1.72 (m, 6H), 3.03–3.18 (m, 2H), 3.24 (t, *J* = 5.41 Hz, 4H), 3.33–3.44 (m, 2H), 3.91 (s, 3H), 7.07 (s, 1H), 7.42 (s, 1H), 7.61 (s, 1H). ^13^C NMR (100 MHz, CDCl_3_) *δ* 11.03, 11.43, 20.67, 21.89, 24.13, 25.52, 46.28, 49.95, 50.66, 52.15, 116.20, 117.06, 117.14, 131.12, 138.55, 147.31, 166.99, 171.08. HRMS (ESI/QTOF) *m*/*z*: [M]^+^ calcd for C_20_H_30_N_2_O_3_ 346.2256; found 346.2242.

#### 3.1.10. 3-(Dipropylcarbamoyl)-5-(piperidin-1-yl)benzoic acid (14)^16^

The mixture of methyl ester 7 (1.19 g, 3.45 mmol), NaOH (0.20 g, 5.19 mmol), MeOH (60 ml), and H_2_O (6 ml) was allowed to stir at 50 °C for 18 h. The reaction was cooled and the MeOH was removed *in vacuo*. The remaining residue was dissolved in 75 ml H_2_O, acidified using 1 M HCl to pH ∼ 4, and extracted with AcOEt (3 × 80 ml). The combined organic extracts were washed with brine, dried over Na_2_SO_4_, filtered, and concentrated *in vacuo*. The residue obtained was purified by FSCC using (EtOH/DCM, v/v, 1/20) as an eluent mixture to give 14 as a white solid (0.66 g, 59%). ^1^H-NMR (100 MHz, CDCl_3_) *δ* 0.68–0.81 (m, 3H), 0.93–1.05 (m, 3H), 1.44–1.55 (m, 2H), 1.52–1.66 (m, 2H), 1.61–1.73 (m, 6H), 3.10–3.22 (m, 2H), 3.22 (t, *J* = 5.52 Hz, 4H), 3.35–3.55 (m, 2H), 7.17–7.32 (m, 1H), 7.40–7.52 (m, 1H), 7.59–7.70 (m, 1H). ^13^C NMR (100 MHz, CDCl_3_) *δ* 11.03, 11.43, 20.64, 21.86, 25.13, 29.70, 46.50, 50.82, 118.28, 119.19, 130.71, 138.20, 169.72, 171.06. HRMS (ESI/QTOF) *m*/*z*: [M + Na]^+^ calcd for C_19_H_28_N_2_O_3_Na 355.1998; found 355.2003.

#### 3.1.11. 2-(3-Methoxyphenyl)propan-2-amine (16)^16^

A suspension of CeCl_3_ (8.67 g, 35.17 mmol) in anhydrous THF (75 ml) was allowed to stir at RT for 1 h. The suspension was cooled to −78 °C and MeLi (22.27 ml, 35.7 mmol, 1.6 M in Et_2_O) was added dropwise. The organocerium complex was allowed to form for a period of 1 h and a solution of 3-methoxy-benzonitrile 15 (1.44 ml, 11.7 mmol) in THF (6 ml) was added dropwise. The reaction mixture was warmed up to RT and allowed to stir for 10 h. The mixture was cooled to 0 °C and quenched with a few drops of aq. NH_4_Cl and 30% NH_4_OH (24 ml) were added dropwise until the precipitate was formed and settled to the bottom of the flask. The mixture was filtered through a pad of Celite and the resulting filtrate was concentrated under reduced pressure. The residue was extracted with Et_2_O washed with brine, dried over MgSO_4_, and then concentrated under reduced pressure. The crude residue was purified by FSCC (eluent 20% MeOH in DCM) to give 16 (4.35 g, 75%) colorless oil. IR (KBr) *ν* (cm^−1^) 3372, 2966, 1610, 1480, 1254, 1038, 704; ^1^H NMR (400 MHz, CDCl_3_) *δ* 1.51 (s, 6H), 3.83 (s, 3H), 6.77–6.80 (m, 1H), 7.08–7.80 (m, 2H), 7.25–7.29 (m, 1H). ^13^C NMR (100 MHz, CDCl_3_) *δ* 32.51, 52.61, 55.23, 111.33, 117.15, 129.32, 152.05, 159.53. HRMS (ESI/QTOF) *m*/*z*: [M + H]^+^ calcd for C_10_H_15_NOH 166.1232; found 166.1222.

#### 3.1.12. ((*R*)-1-(((Benzyloxy)carbonyl)amino)-2-phenylethyl)(((2-(3-methoxyphenyl)propan-2-yl) amino)methyl)phosphinic acid (18)

1-(3-Methoxy-phenyl)-1-methyl-ethylamine 16 (0.45 mmol, 70 mg), (*R*)-17 (0.30 mmol, 95 mg) and paraformaldehyde (0.60 mmol, 13 mg) were added to a Schlenk tube, then, the tube was charged with argon. Toluene (2 ml) was added using a syringe. The reaction mixture was allowed to stir at 110 °C until the substrate disappeared as determined by TLC. After cooling to RT, the reaction system was purified by FSCC (CHCl_3_/MeOH/AcOH, v/v, 7/0.5/0.5) to afford pure 18 as a white solid (71.6 mg, 48%). ^1^H NMR (400 MHz, CD_3_OD) *δ* 1.21 (s, 6H), 1.76 (d, *J* = 6.14 Hz, 1H), 1.85 (s, 1H), 2.69–2.84 (m, 1H), 3.83 (s, 3H), 3.90–3.94 (m, 1H), 4.10 (t, *J* = 6.54 Hz, 3H), 4.99 (s, 2H), 6.95–7.46 (m, 17H); ^13^C NMR (100 MHz, CD_3_OD) *δ* 26.78, 27.78, 35.93, 49.56, 54.49, 60.74, 65.84, 110.81, 112.72, 117.20, 119.31, 125.74, 126.94, 128.28, 129.32, 130.21, 130.88, 136.81, 138.96, 143.36, 156.91, 160.28, 163.33; ^31^P NMR (162 MHz, CD_3_OD) *δ* 39.69. HRMS (ESI/QTOF) *m*/*z*: [M + Na]^+^ calcd for C_27_H_33_N_2_O_5_PNa 519.2025; found 519.2023.

#### 3.1.13. ((*R*)-1-Amino-2-phenylethyl)(((2-(3-methoxyphenyl)propan-2-yl)amino)methyl)phosphinic acid (19)

In a 50 ml dry round-bottom flask, 135 mg of 10% Pd/C was added to MeOH (4 ml) of 18 (0.5 mmol, 200 mg). The flask was purged several times with H_2_, and the reaction was allowed to stir for 24 h. The Pd/C was filtered, the solvent was evaporated under reduced pressure then the desired compound 19 was obtained quantitatively as a white solid (181 mg, 99%). ^1^H NMR (400 MHz, CD_3_OD) *δ* 1.28 (s, 6H), 1.75 (d, *J* = 6.21 Hz, 1H), 1.76 (s, 1H), 2.68–2.81 (m, 1H), 3.86 (s, 3H), 3.92 (m, 1H), 4.00 (t, *J* = 7.31 Hz, 3H), 6.94–7.61 (m, 11H); ^13^C NMR (100 MHz, CD_3_OD) *δ* 22.47, 27.64, 36.43, 49.65, 54.78, 60.26, 111.38, 113.40, 117.17, 119.03, 120.36, 126.42, 129.16, 130.26, 137.80, 143.20, 160.09, 163.58; ^31^P NMR (162 MHz, CD_3_OD) *δ* 17.60. HRMS (ESI/QTOF) *m*/*z*: [M + NH_4_]^+^ calcd for C_19_H_27_N_2_O_3_PNH_4_ 380.2103; found 380.2100.

#### 3.1.14. ((*R*)-1-(3-(Dipropylcarbamoyl)-5-(piperidin-1-yl)benzamido)-2-phenylethyl)(((2-(3-methoxy phenyl)propan-2-yl)amino)methyl)phosphinic acid (2)

In a dry flask under Ar, a mixture of HATU (380 mg, 1.0 mmol) and acid 14 (330 mg, 1.0 mmol) in DMF (0.6 ml/600 µl) was allowed to stir for 30 min. Amine 19 (360 mg, 1.0 mmol) and 4-ethylmorpholine (0.19 ml/190 µl, 1.5 mmol) were added, and the reaction was allowed to stir at RT for 24 h. The reaction mixture was quenched with sat. NaHCO_3(aq.)_ and extracted with AcOEt. The organic layer was dried over anhydrous Na_2_SO_4_, and the solvent was removed under reduced pressure. The residue obtained was purified by FSCC using AcOEt/MeOH/NH_4_OH_(aq.)_ (25%), v/v, 20/1/0.01, to afford product 2 (400 mg, 58%) as yellow waxy solid. ^1^H NMR (400 MHz, CDCl_3_) *δ* 0.80 (t, *J* = 6.85 Hz, 3H), 0.95 (t, *J* = 6.85 Hz, 3H), 1.21 (s, 3H), 1.30 (s, 3H), 1.41–1.50 (m, 2H), 1.55–1.62 (m, 2H), 1.66–1.80 (m, 6H), 2.74 (d, *J* = 5.01 Hz, 2H), 2.90–3.00 (m, 1H), 3.06–3.09 (m, 1H), 3.11–3.17 (t, *J* = 6.83 Hz, 2H), 3.28–3.32 (m, 2H), 3.40–3.49 (m, 2H), 3.60–3.67 (m, 1H), 3.75–3.80 (d, *J* = 12.21 Hz, 1H), 3.81 (s, 3H), 3.84–3.92 (d, *J* = 12.41 Hz, 1H), 4.20–4.31 (m, 1H), 5.20 (s, 1H), 6.52–6.64 (d, *J* = 8.02 Hz, 1H), 6.75–6.83 (m, 1H), 6.88–7.32 (m, 11H). ^13^C NMR (100 MHz, CDCl_3_) *δ* 13.83, 14.72, 19.25, 26.35, 27.77, 29.70, 30.25, 41.14, 47.47, 55.64, 66.35, 67.49, 68.46, 71.98, 112.33, 114.17, 118.11, 128.12, 129.54, 129.75, 130.09, 130.89, 136.03, 137.86, 138.92, 143.51, 149.01, 153.23, 156.75, 156.75, 159.93; ^31^P NMR (162 MHz, CDCl_3_) *δ* 25.34. HRMS (ESI/QTOF) *m*/*z*: [M]^+^ calcd for C_38_H_53_N_4_O_5_P 676.3754; found 676.3723.

### 3.2. Biological evaluation

#### 3.2.1. Antiplasmodial activity

To assess the antiplasmodial activity against erythrocytic-stage *P. falciparum*, synchronized parasites (with 5% sorbitol) were incubated with test compounds at 3-fold dilutions, ranging from 10 µM to 0.5 nM. This incubation occurred in 96-well plates at 37 °C for 48 hours. The culture medium consisted of RPMI-1640 supplemented with 25 mM HEPES (pH 7.4), 0.5% Albumax, and 100 µM hypoxanthine, within an atmosphere of 3% O_2_, 5% CO_2_, and 91% N_2_. Post-incubation, cells were fixed in 2% formaldehyde in phosphate-buffered saline (PBS) and transferred into PBS with 100 mM NH_4_Cl, 0.1% Triton X-100, and 1 nM YOYO-1. Parasitemia was determined through dot plots (forward scatter *versus* fluorescence) acquired using a FACSort flow cytometer and the data were analyzed with CellQuest software (Becton Dickinson). Calculation of IC_50_ values, derived from comparisons with untreated control cultures, was performed using GraphPad PRISM software. Two independent experiments were conducted, each with two replicates for every set of experimental conditions.

### 3.3. *In silico* evaluation of ADMET

The pharmacokinetic properties including absorption, distribution, metabolism, excretion and toxicity parameters (ADMET) of compound 1 and compound 2 were estimated using pkCSM.^38–42^

### 3.4. Molecular modelling and simulation

#### 3.4.1. Molecular docking

The 3D coordinates of the molecular target, plasmepsin II (PDB: 4CKU) was obtained from the Protein Data Bank. Molecular interactions of compound 1 and compound 2 with plasmepsin II were evaluated using the XP scoring function in Glide (Schrödinger Suite 2023.1).^43^ The grid box was created with a size of 20 Å around the corresponding co-crystallized ligand. The complex with the most favorable docking score (lowest GScore) from the Glide results was selected for subsequent molecular dynamics simulations. Visualization of the docked complexes was conducted by utilizing the PyMOL Molecular Graphics System^44,45^ and Discovery Studio Visualizer (BIOVIA Dassault Systems, Pharmacopeia, Inc.).^46,47^

#### 3.4.2. Molecular dynamics simulation

MD simulations were performed to investigate the unbound form of plasmepsin II and its complex with compound 1 and compound 2 using the GROMACS 2023.2 package^48–51^ for a duration of 300 ns. Topology parameters for the ligands were generated using the ACPYPE.^52^ The AMBER99SB-ILDN force field^53^ was used for the system, while water molecules were described using the TIP3P model. Each system was solvated in a cubic periodic box with a minimum distance of 1.5 nm between the protein and the box edge. Na^+^ and Cl^−^ ions were added to a physiological concentration of 0.15 M to neutralize the system, followed by energy minimization to optimize atomic coordinates.^54^ Subsequently, the system underwent energy minimization using the steepest descent algorithm followed by conjugate gradient optimization. Production-phase trajectories were generated over 300 ns under isothermal conditions (310.15 K).^55^ The stability and flexibility of each system were evaluated from the equilibrium phase of the trajectories in accordance with previously reported methodologies by calculating root-mean-square deviation (RMSD), root-mean-square fluctuation (RMSF), radius of gyration (*R*_g_), solvent-accessible surface area (SASA), and protein–ligand hydrogen-bonding interactions.^56–60^

#### 3.4.3. MM/PBSA binding free energy calculation

Binding free energies (Δ*G*) were computed using the Molecular Mechanics/Poisson–Boltzmann Surface Area (MM/PBSA)^47^ approach implemented in the *gmx_MMPBSA* tool.^56^ For the MM/PBSA analysis, the 200–300 ns portion of each trajectory, were selected to represent the equilibrated stage of the simulation.^61^ The binding free energy in the condensed phase is given by the following equation:Δ*G* = Δ*G*_gas_ + Δ*G*_solv_ − *T*Δ*S*Δ*G*_gas_ represents the gas-phase binding energy, which comprises the van der Waals interactions (*E*_VDWAALS_) and the electrostatic energy in vacuum (*E*_EEL_); Δ*G*_solv_ denotes the change in solvation free energy upon binding, which includes the polar solvation contribution (calculated by solving the Poisson–Boltzmann equation, *E*_EPB_) and the non-polar solvation energy (estimated based on the solvent-accessible surface area, *E*_ENPOLAR_); −*T*Δ*S* accounts for the change in conformational entropy upon binding, where *T* is the simulation temperature and Δ*S* is the entropy change.

#### 3.4.4. Principal component analysis

Principal component analysis (PCA) was applied to characterize the dominant collective motions within the plasmepsin II binding pocket. The analysis was conducted on the backbone atoms of key residues defining the binding pocket. The covariance matrix of atomic fluctuations was constructed using the *gmx covar* module in GROMACS to extract the principal components (PCs) and eigenvalues. The resulting conformational ensembles were projected onto the first two principal components (PC1 and PC2) *via* the *gmx anaeig* module. Finally, the Gibbs free energy landscape (FEL) was constructed from the probability distribution of the PC1 and PC2 projections using the *gmx sham* module. PCA was performed using the complete 300 ns trajectory, and the same trajectory range was used for the subsequent FEL analysis based on PC1 and PC2.

## 4. Conclusions

In conclusion, this study describes the design and synthesis of a novel phosphinic acid-based bioisostere of the hydroxyethylamine motif found in plasmepsin inhibitor scaffolds. The successful preparation of compound 2 and its evaluation against the drug-resistant *P. falciparum* W2 strain demonstrated that phosphinic acid replacement can retain measurable antiplasmodial activity.

Computational investigations, including molecular docking, 300 ns molecular dynamics simulations, and MM/PBSA analysis, provided insights into the potential binding mode and interaction profile of compound 2 within the plasmepsin II active site. These results support the use of phosphinic acid as an alternative scaffold for further exploration in plasmepsin-focused antimalarial research.

However, the current findings represent an early-stage scaffold evaluation. Further studies involving cytotoxicity assessment, selectivity index determination, enzymatic validation of plasmepsin II inhibition, and structural optimization will be necessary to define the full biological potential of this scaffold.

Overall, compound 2 provides a useful starting point for future medicinal chemistry efforts aimed at improving antiplasmodial potency and drug-like properties.

## Conflicts of interest

The authors declare that there are no conflicts of interest.

## Supplementary Material

RA-OLF-D6RA09362K-s001

## Data Availability

The data supporting this article are available in the supplementary information (SI). Supplementary information: experimental procedures and characterization data. See DOI: https://doi.org/10.1039/d6ra09362k.
