# Supplementary material for: A phosphinic acid bioisostere of hydroxyethylamine as a novel antiplasmodial scaffold: design, synthesis, and in vitro and in silico evaluation
Source: RSC Adv. 2026 Nov 11. Online ahead of print. doi: 10.1039/d6ra09362k (PMC13753973; doi:10.1039/d6ra09362k)
Supplement: Supplementary file 1 [file RA-OLF-D6RA09362K-s001.pdf]

Supplementary Information (SI) for RSC Advances.
This journal is © The Royal Society of Chemistry

Supplementary Data

A Phosphinic Acid Bioisostere of Hydroxyethylamine as a Novel Antiplasmodial Scaffold: Design, Synthesis, and *In Vitro* and *In Silico* Evaluation

Moaz M. Abdou[1,*]; Ahmed I. Khodair[2]; Danwen Qiu[3]; Rasha A. El-Saeed[4]; Nawal Bahtati[5]; Faez Iqbal Khan[6,*]; Magdalini Matziari[3,7,*]

[1]Egyptian Petroleum Research Institute, Nasr City, Cairo 11727, Egypt

[2]Chemistry Department, Faculty of Science, Kafrelsheikh University, 33516, Kafrelsheikh, Egypt

[3]Centre of Excellence for Molecular Innovation and Advanced Materials, Academy of Life and Natural Sciences, Xi'an Jiaotong-Liverpool University, 111 Ren'ai Road, SIP, Suzhou, Jiangsu Province, 215123, P. R. China

[4]Department of Chemistry, Faculty of Science, Mansoura University, Mansoura 31556, Egypt

[5]Chemistry Department, Faculty of Science, Applied Science Private University, Amman, 11931, Jordan

[6]Centre of Excellence for Oncology and Immunology, Academy of Life and Natural Sciences, Xi'an Jiaotong-Liverpool University, 111 Ren'ai Road, SIP, Suzhou, Jiangsu Province, 215123, P. R. China

[7]Department of Chemistry, Section of Organic Chemistry and Biochemistry, University of Ioannina, 45110 Ioannina, Greece

Materials and Instrumentation

All commercially available reagents and starting materials were purchased from Aldrich, Sigma, Novabiochem, Aladdin, TCI and Acros Organics and directly used without further purification. Unless otherwise stated, all non-aqueous reactions were carried out under an atmosphere of dry Ar or using Schlenk-line techniques and all glassware was flame dried prior to use. When necessary, solvents and reagents were dried prior to use. Anhydrous toluene and THF were purchased from Aldrich, purged with argon, and passed through a solvent purification system (PureSolv, Innovative Technology-Amesbury, MA). Other anhydrous solvents were obtained by storing analytical quality grade solvents over 3 Å or 4 Å activated molecular sieves. *i*Pr_2NEt was distilled from CaH_2 and stored over 4 A° molecular sieves under an inert atmosphere prior to use. Column chromatography was carried out using silica gel (40 Å, Acros Organics) as a stationary phase, either using gravity flow or air overpressure flow conditions according to the method of Still.[12] Mobile phases are described in each experiment. Thin layer chromatography (TLC) was performed with alumina plates coated with Merck silica gel 60 F254 (layer thickness: 0.2 mm) and analyzed under UV-light (254 nm) or stained with a potassium permanganate, *p*-anisaldehyde, ninhydrin and PMA stain. Organic layers were routinely dried with anhydrous $MgSO_4$ or Na_2SO_4 and concentrated using a Büchi rotary evaporator. Melting points were determined on an MPA100 OptiMelt (Stanford Research Systems, Sunnyvale, USA) and are reported uncorrected (heating rate 5 °C/min).

^{1}H, ^{31}P and ^{13}C NMR spectra were recorded on a Bruker Avance III 400 (400 MHz for ^{1}H, 100 MHz for ^{13}C and 162 MHz for ^{31}P) at ambient temperature. ^{13}C partial assignment is based on DEPT experiments. ^{13}C and ^{31}P NMR spectra are fully proton decoupled. TMS (^{1}H NMR), and 85% H_3PO_4 (^{31}P NMR) were used as internal references. $CDCl_3$, Methanol-d_4, and DMSO-d_6 were used as solvents. Chemical shifts are reported in delta (δ) units, parts per million (ppm) relative to the residual signals of the deuterated solvents as the internal standard. The coupling constants (*J*) are reported in Hz and signal multiplicities are reported as (s = singlet, d = doublet, t = triplet, q = quartet, br = broad, m = multiplet). Assignments were supported by DEPT for almost compounds. For mass spectrometry data acquisition, a micrOTOF-Q II electrospray time-of-flight (ESI-TOF) mass spectrometer (Bruker Daltonik GmbH, Bremen, Germany) was used; this was coupled to an Agilent 1260 LC system (Agilent Technologies, Waldbronn, Germany). Negative or positive ion ESI spectra were acquired by adjusting the needle and cone voltages accordingly.. Infrared spectra (IR) were recorded on Agilent Cary 660 FTIR spectrometer and were taken as thin films or as KBr discs and only the most representative frequencies (in cm^{-1}) are given. RP-HPLC analysis of the final compound **2** carried out on an Agilent Eclipse XDB-C_{18} column (4.6 X 250 nm), which thermostated at 27 °C. The flow rate was 0.4 ml/min, whereas the injection volume was 15 uL. The final compounds **2** was demonstrated to have 98% purity.

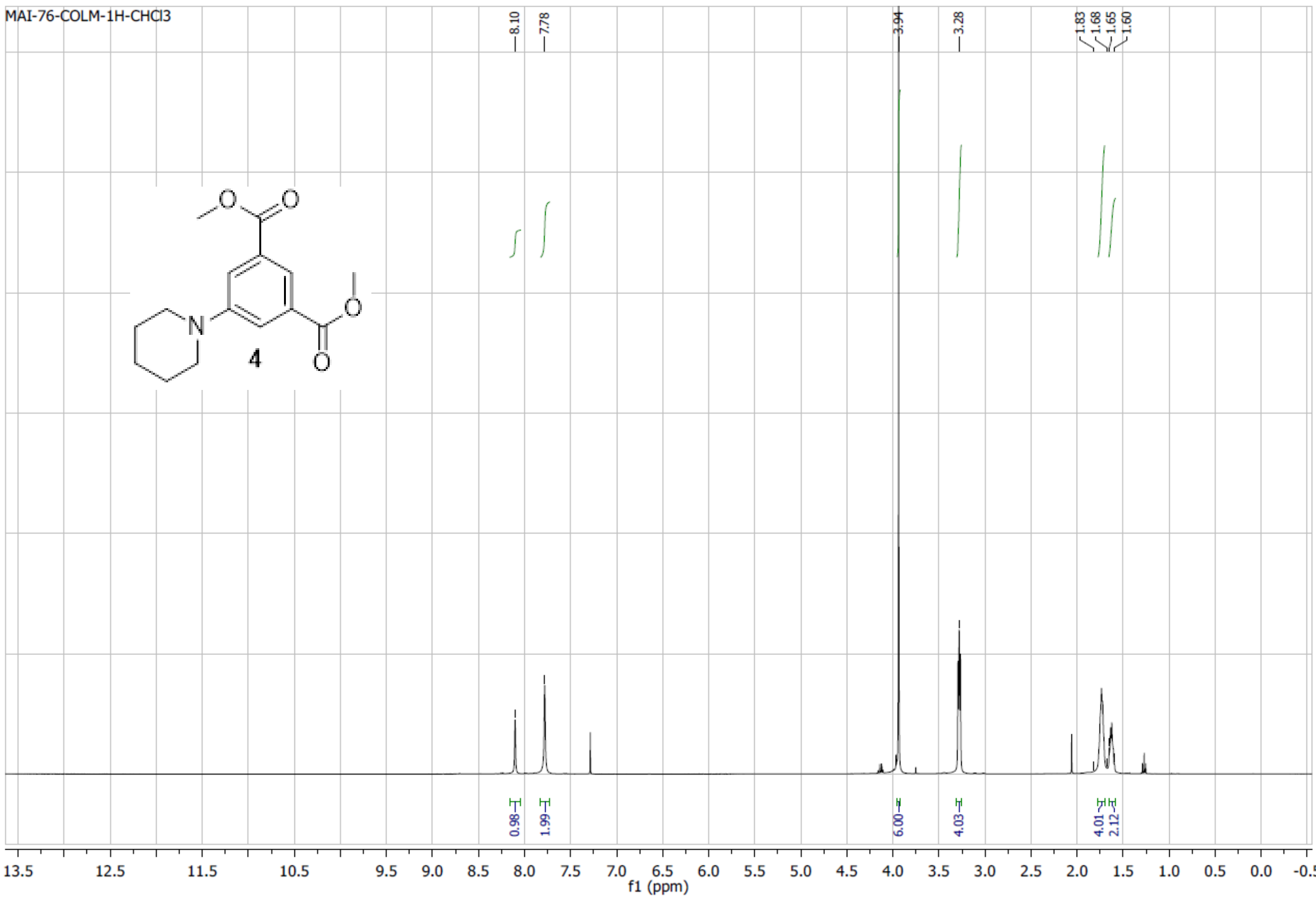

Figure S1. ^{1}H-NMR spectrum of **4**

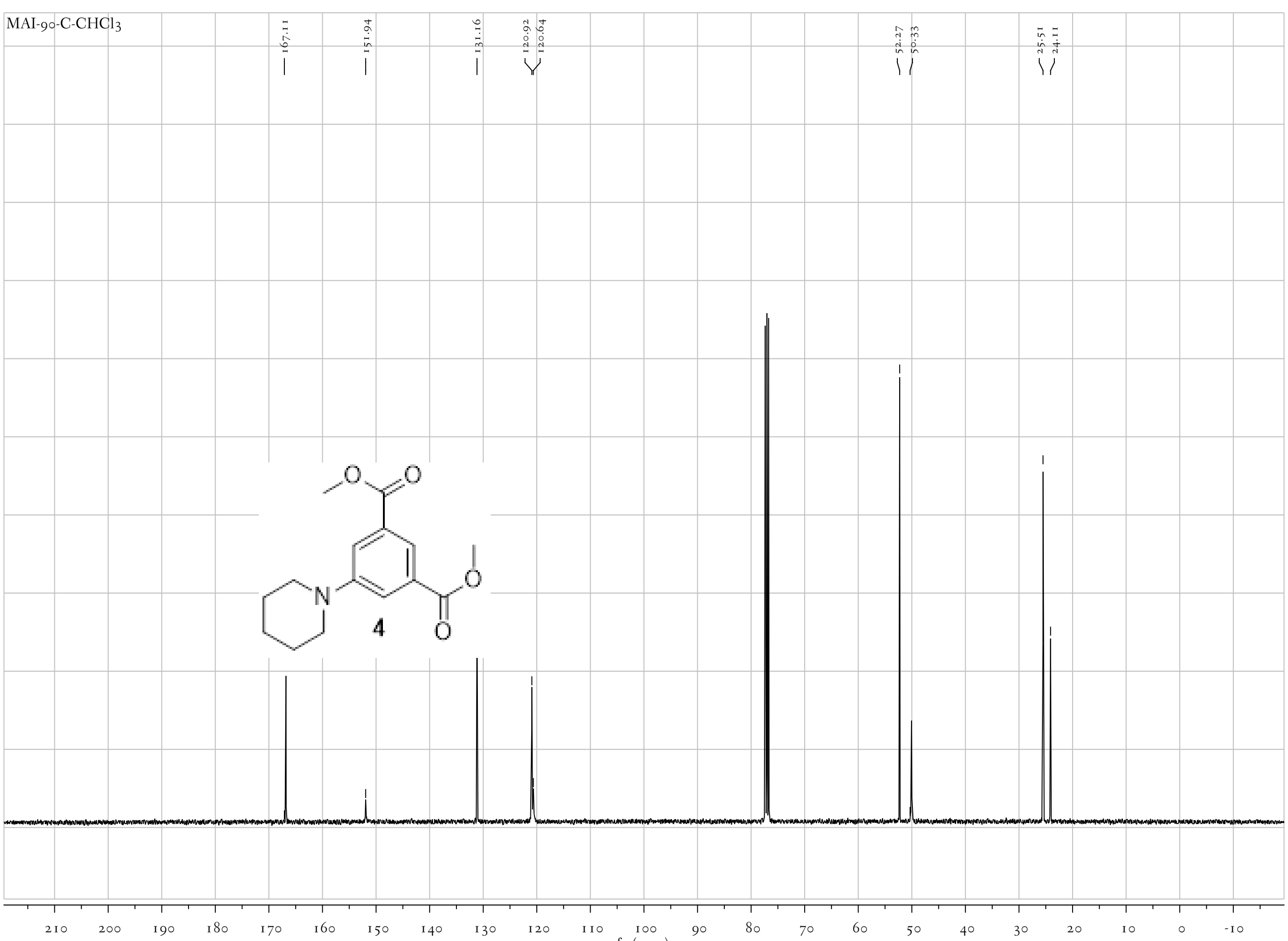

Figure S2. ^{13}C-NMR spectrum of **4**

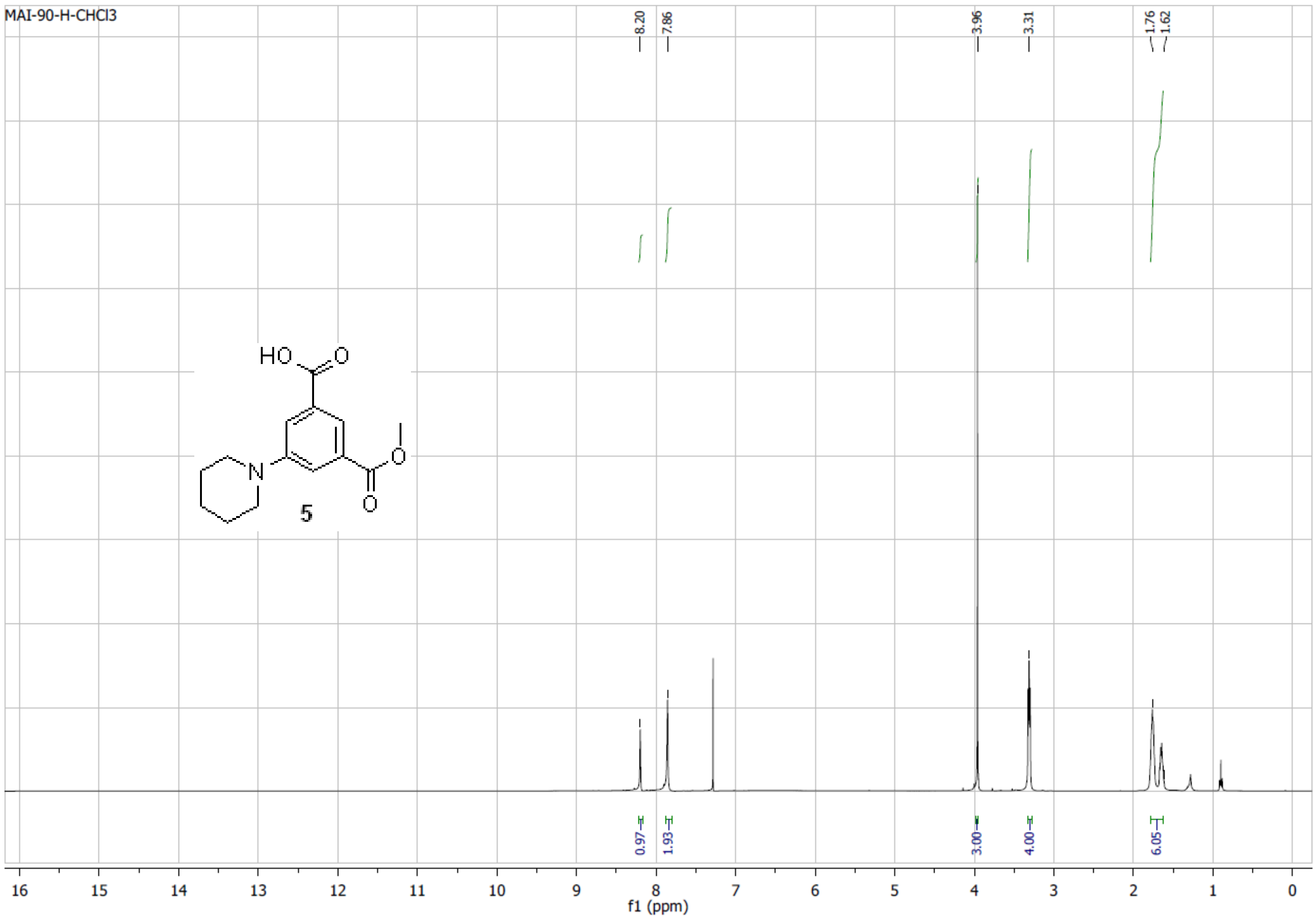

Figure S3. ^{1}H-NMR spectrum of **5**

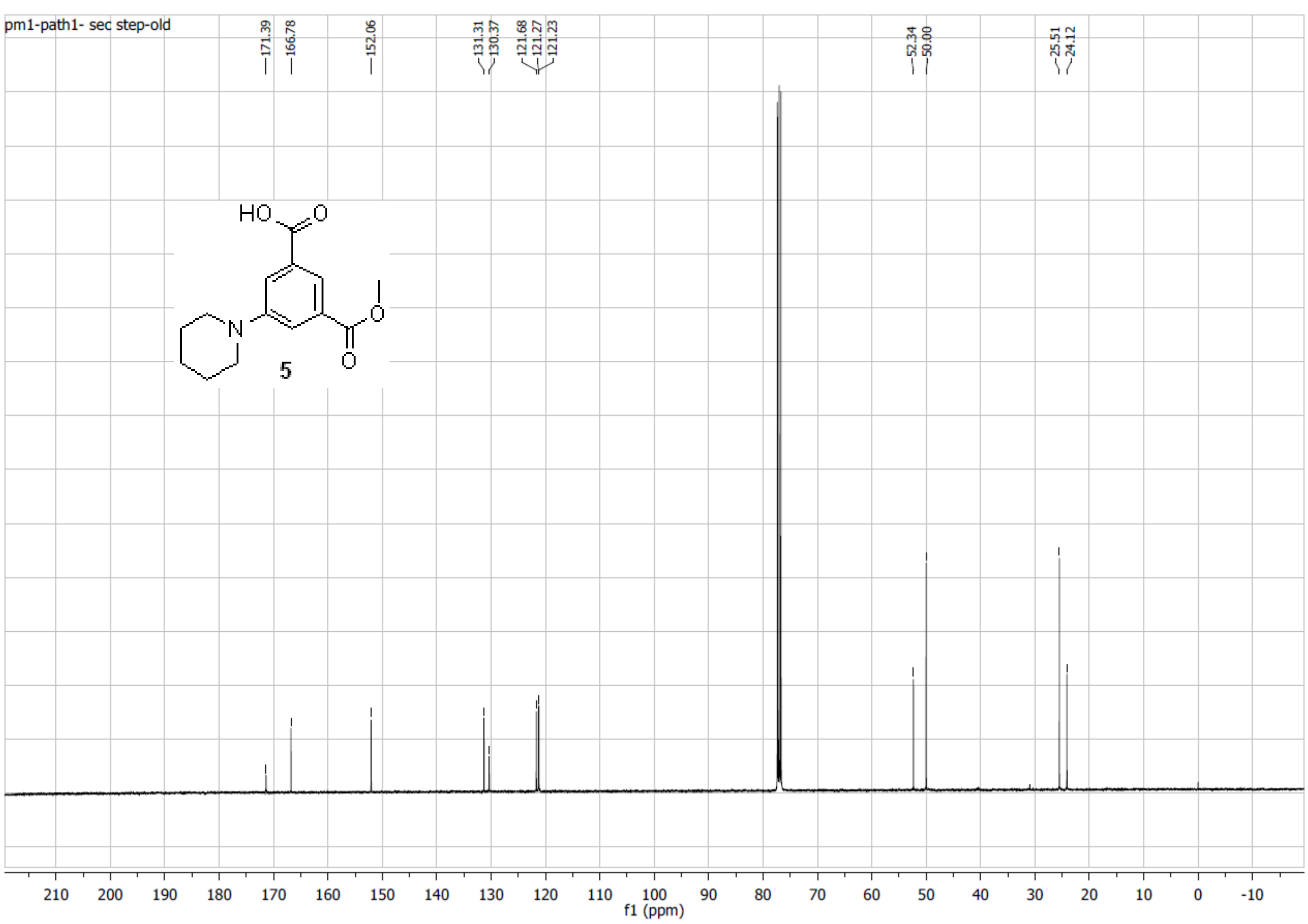

Figure S4. ^{13}C-NMR spectrum of **5**

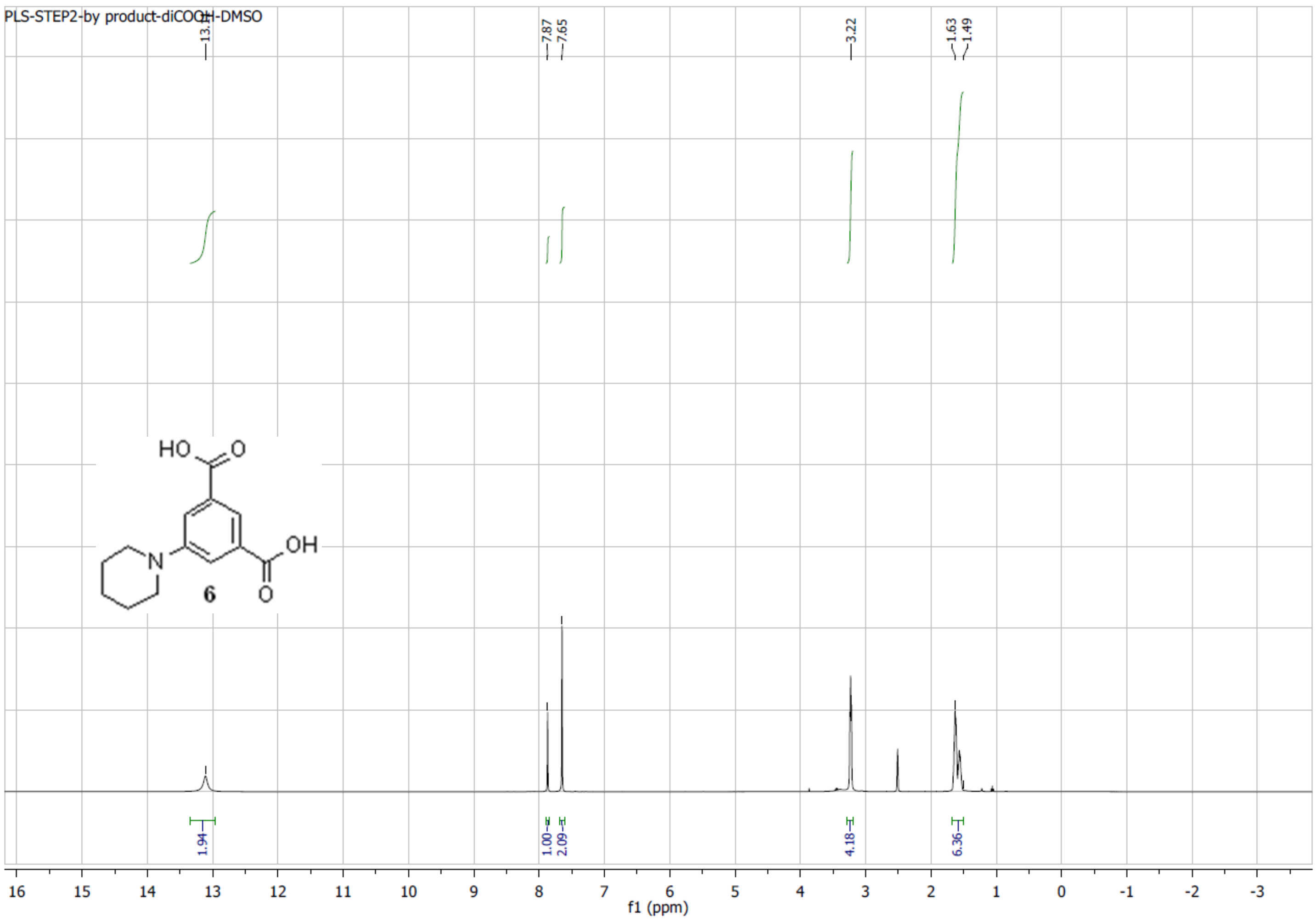

Figure S5. ^{1}H-NMR spectrum of **6**

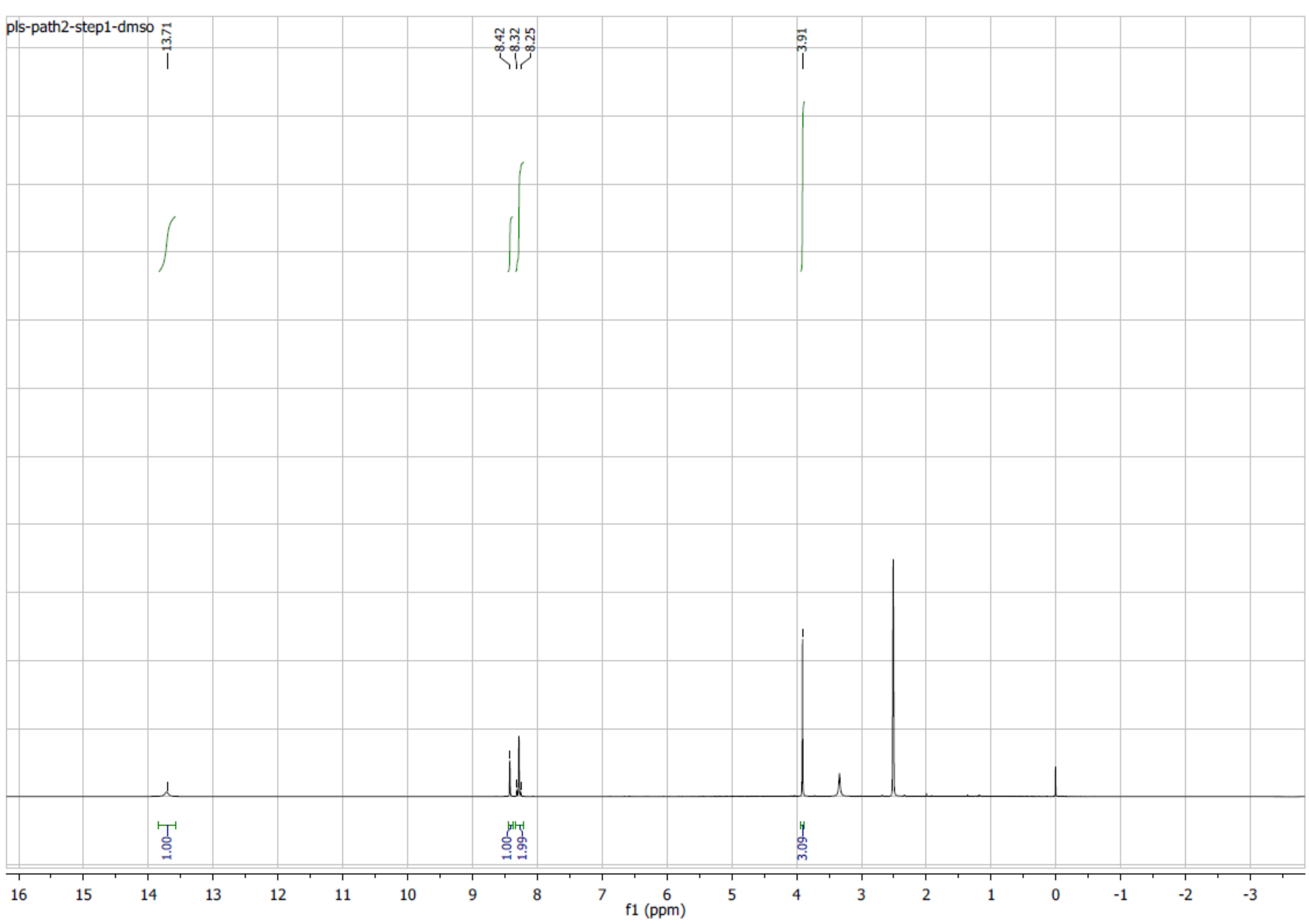

Figure S6. ^{13}C-NMR spectrum of **6**

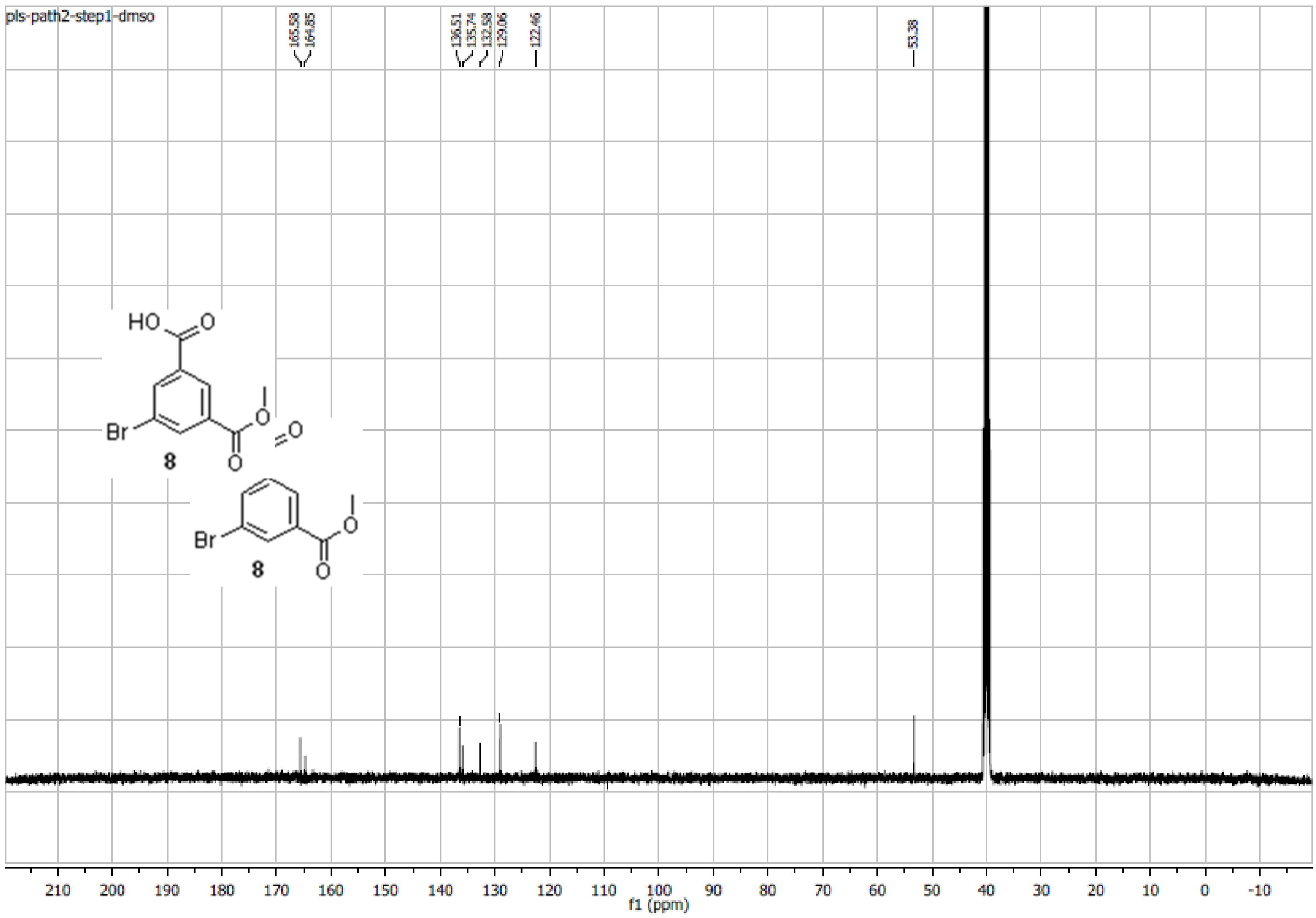

Figure S7. ^{13}C-NMR spectrum of **8**

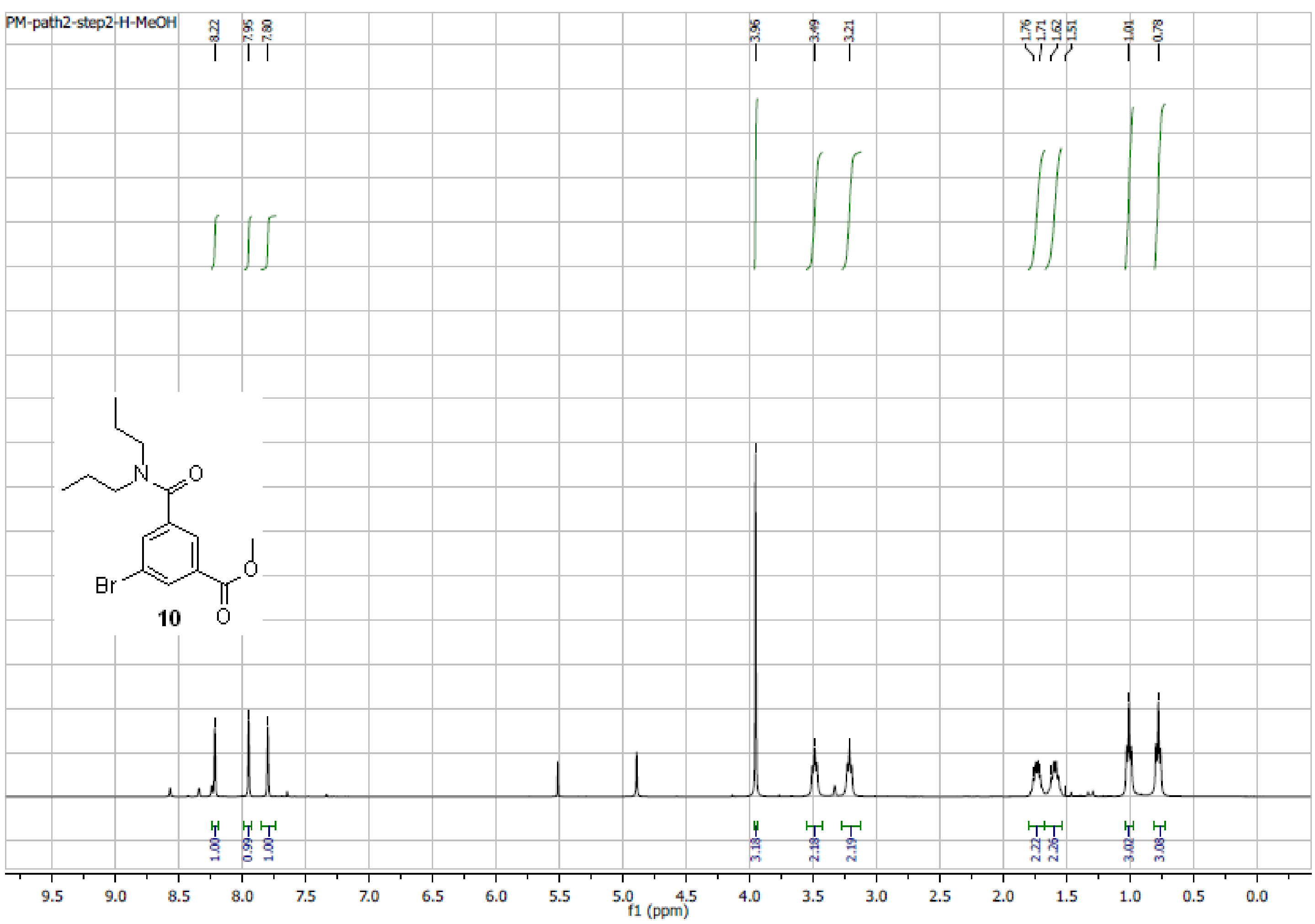

Figure S8. ^{1}H-NMR spectrum of **10**

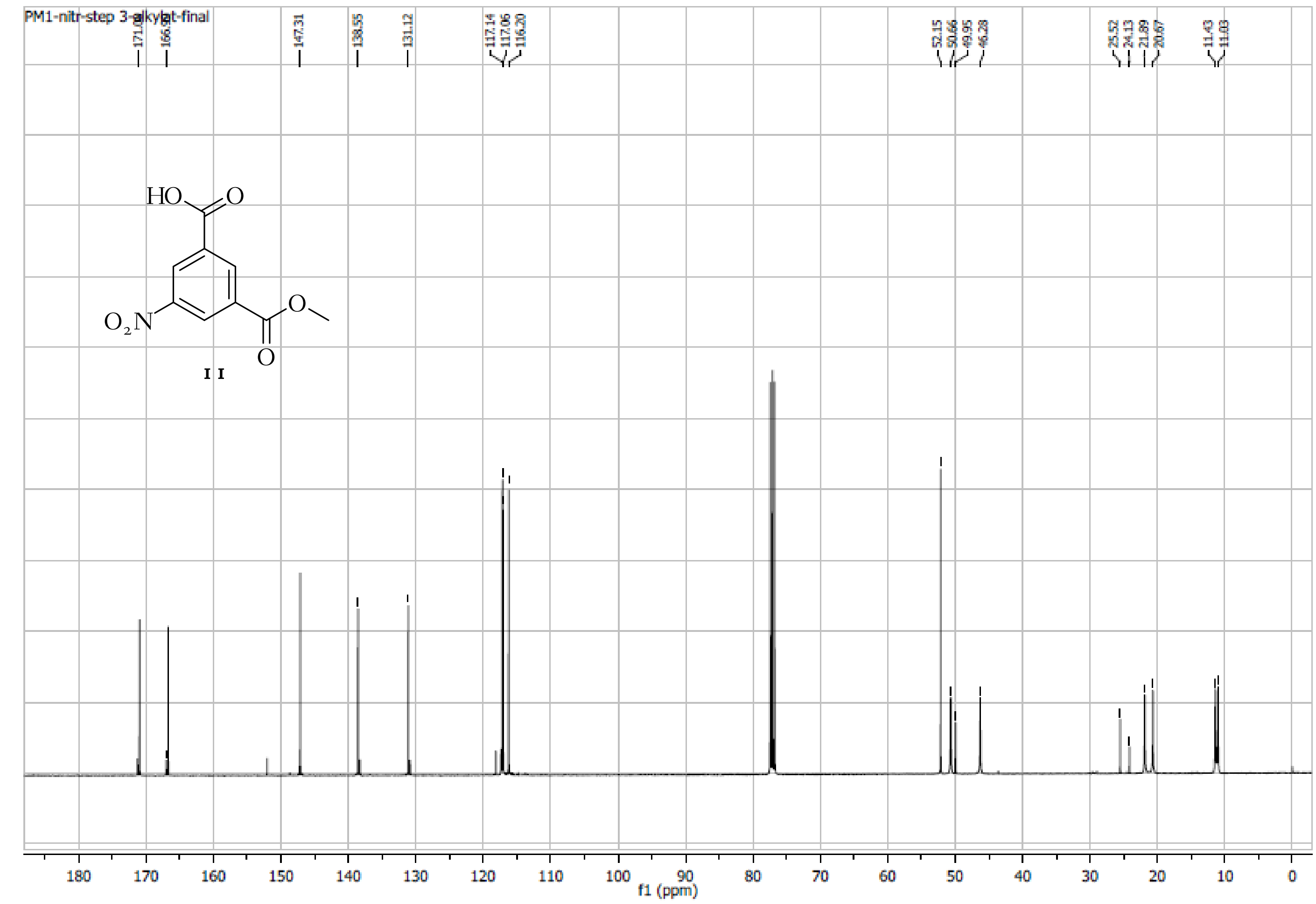

Figure S9. ^{13}C-NMR spectrum of **11**

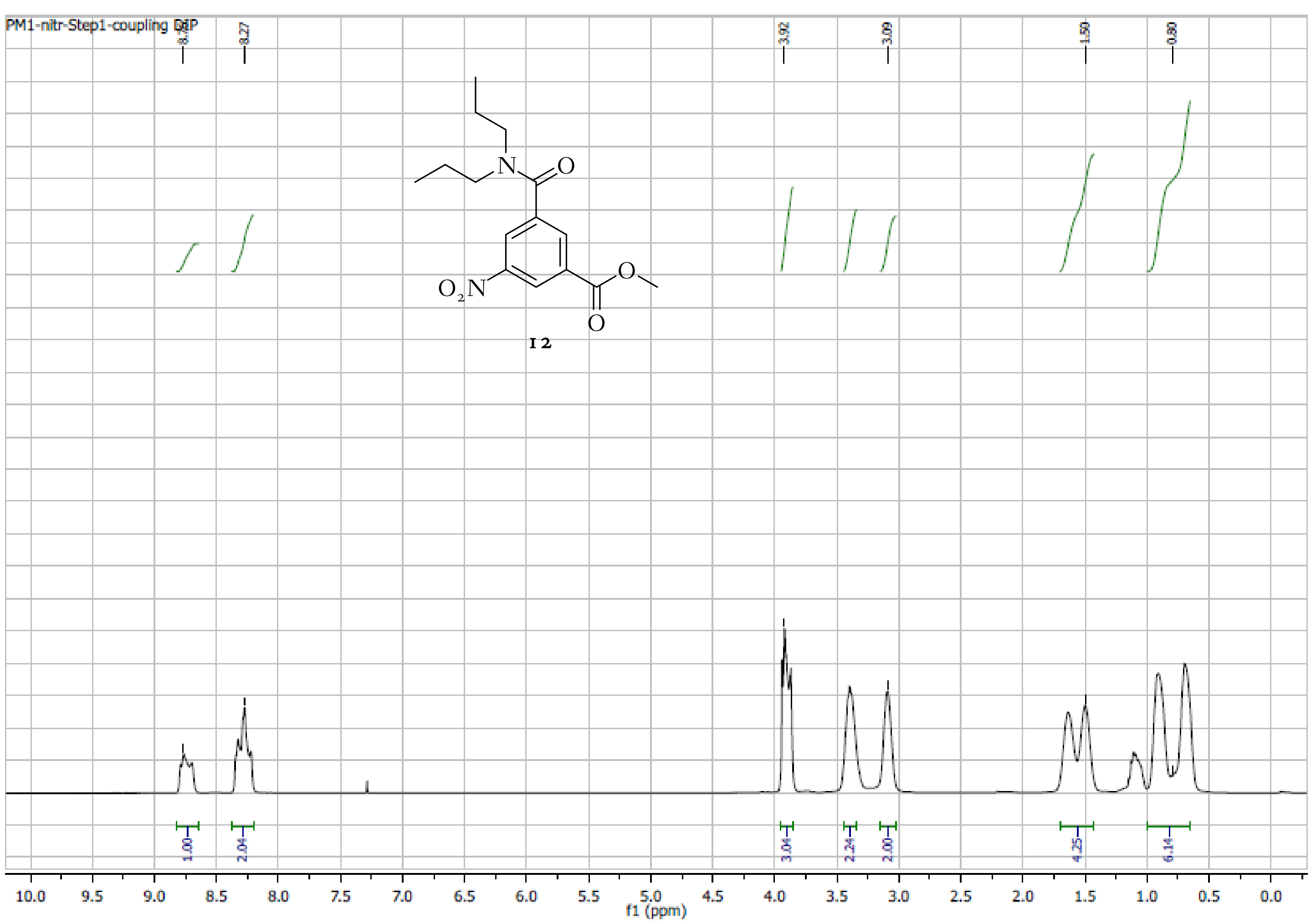

Figure S10. ^{1}H-NMR spectrum of **12**

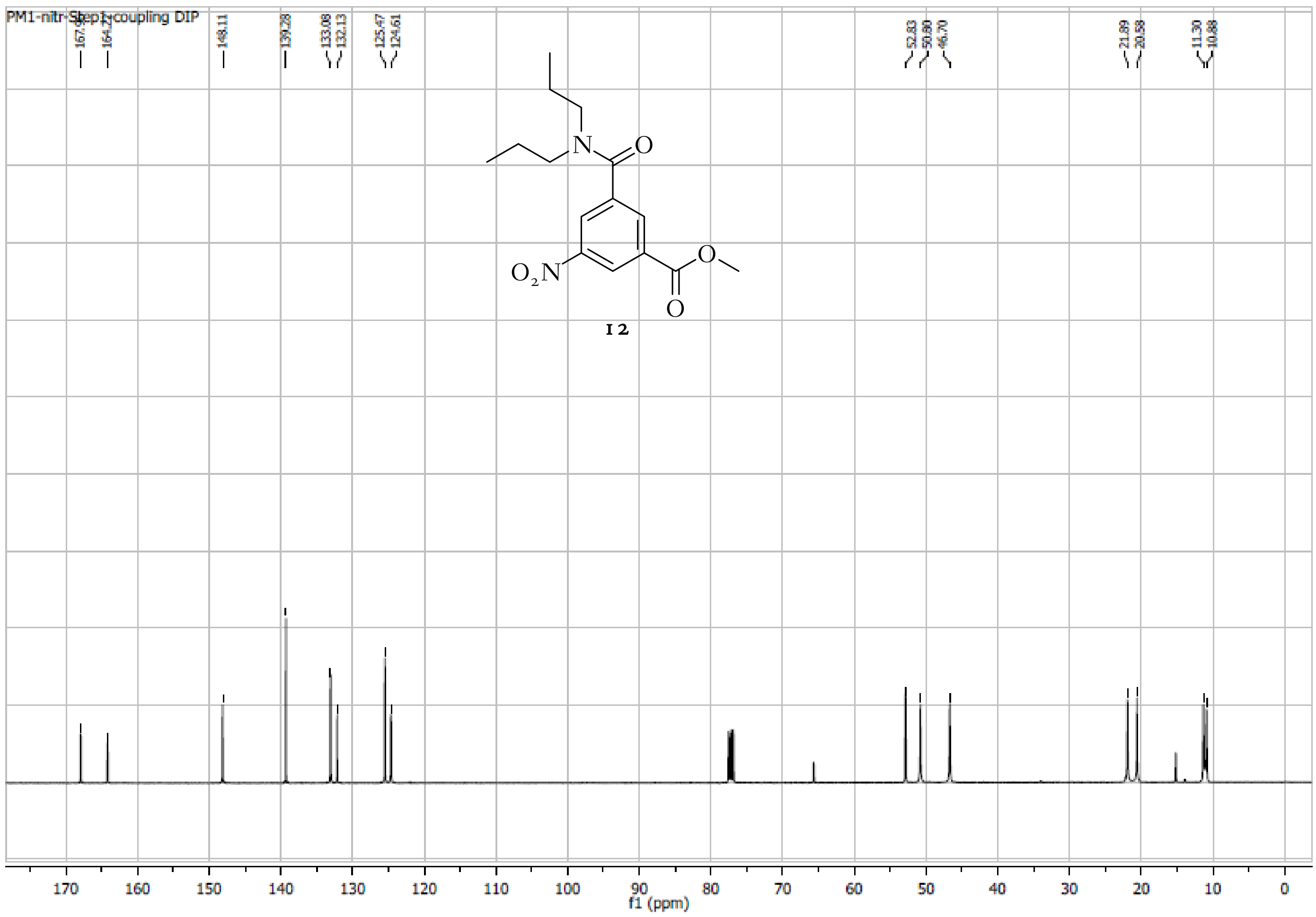

Figure S11. ^{13}C-NMR spectrum of **12**

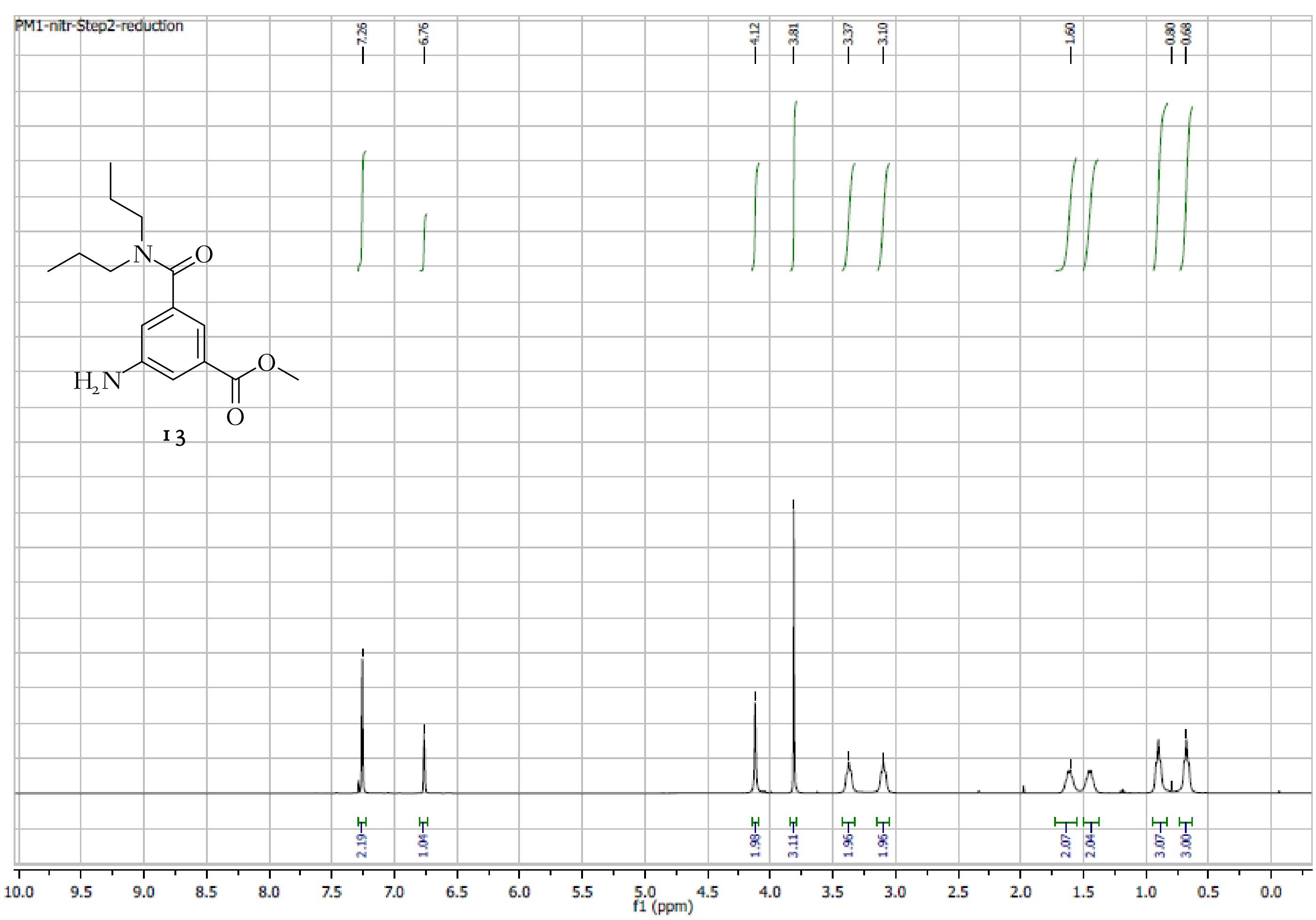

Figure S12. ^{1}H-NMR spectrum of **13**

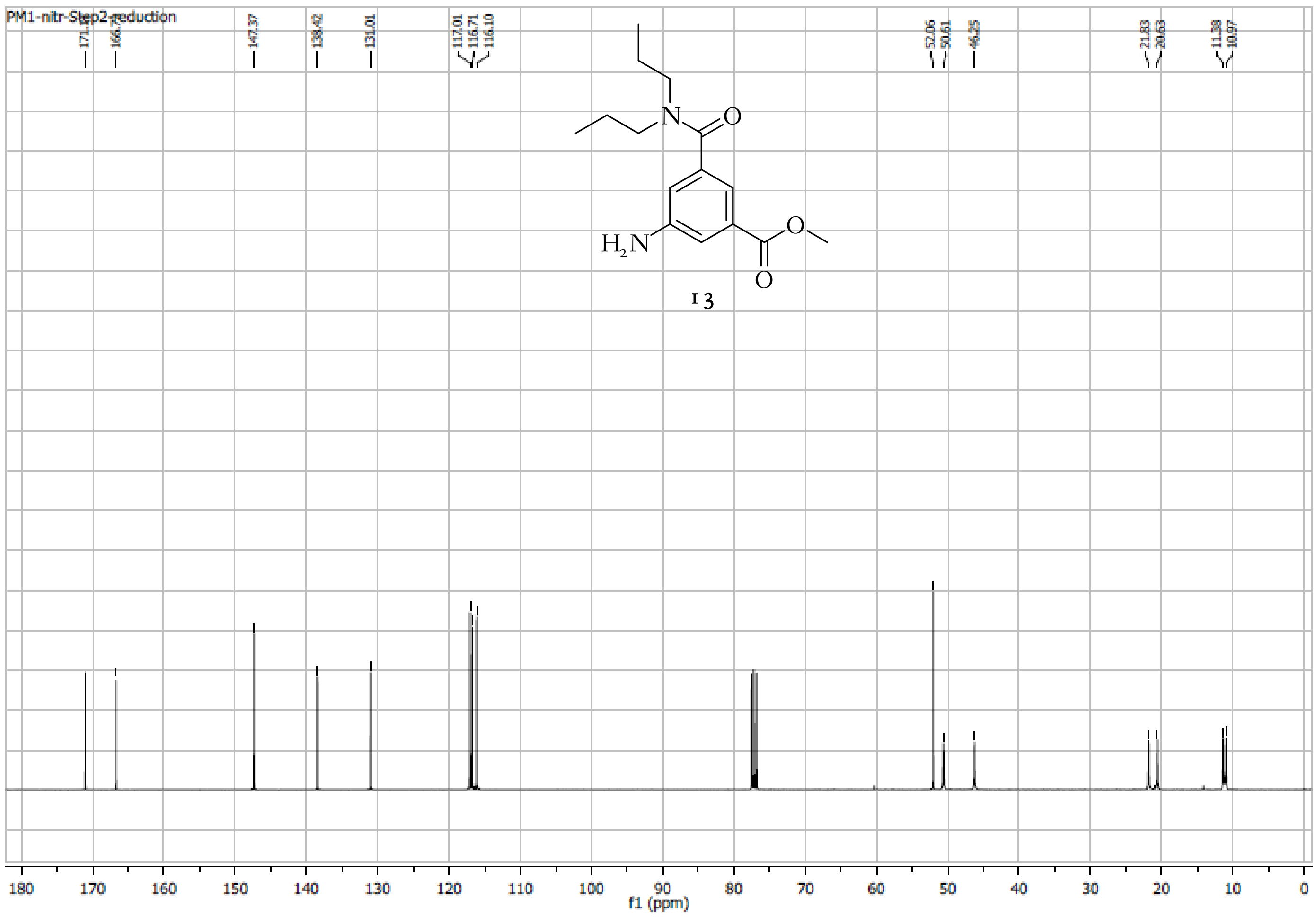

Figure S13. ^{13}C-NMR spectrum of **13**

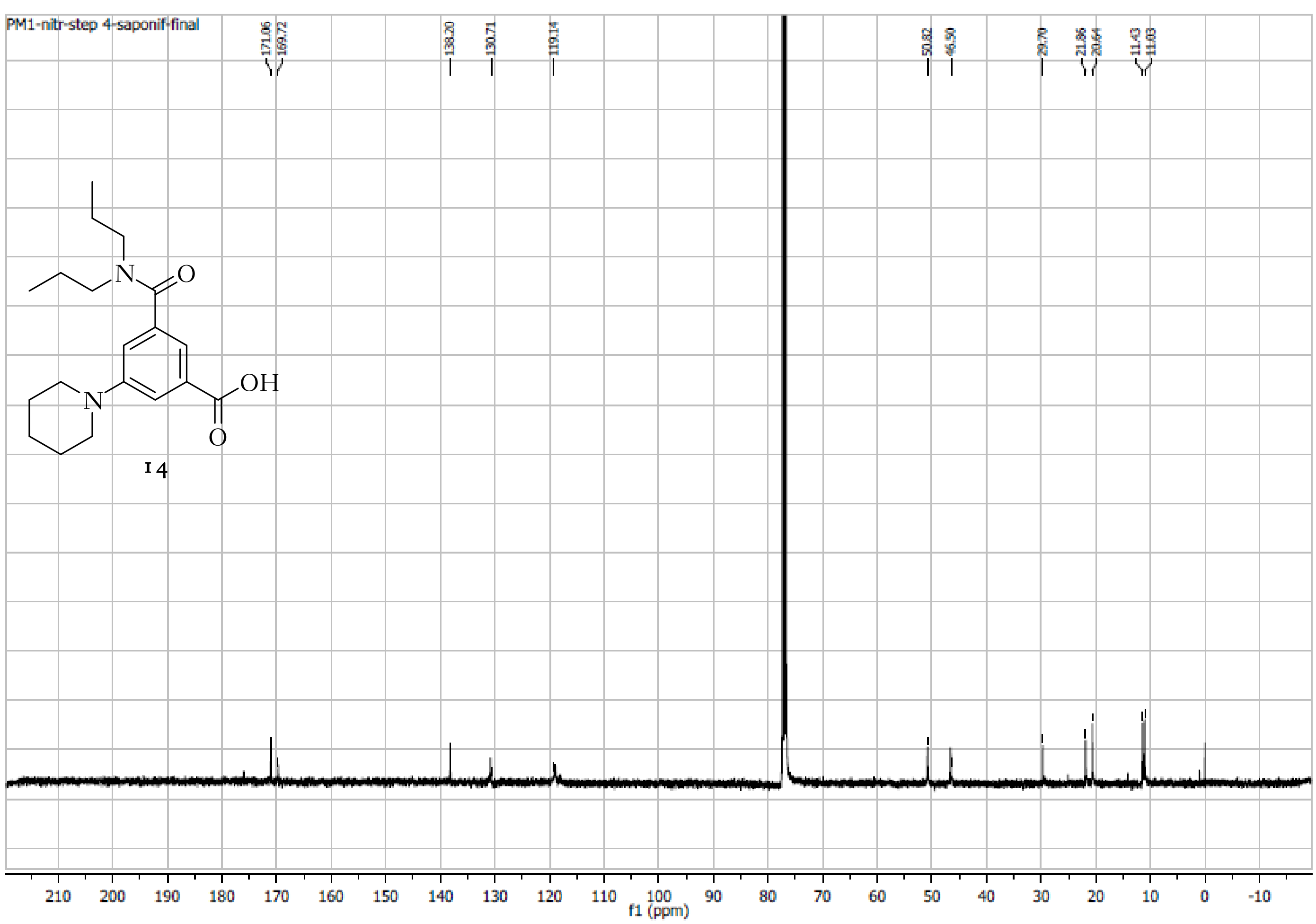

Figure S14. ^{13}C-NMR spectrum of **14**

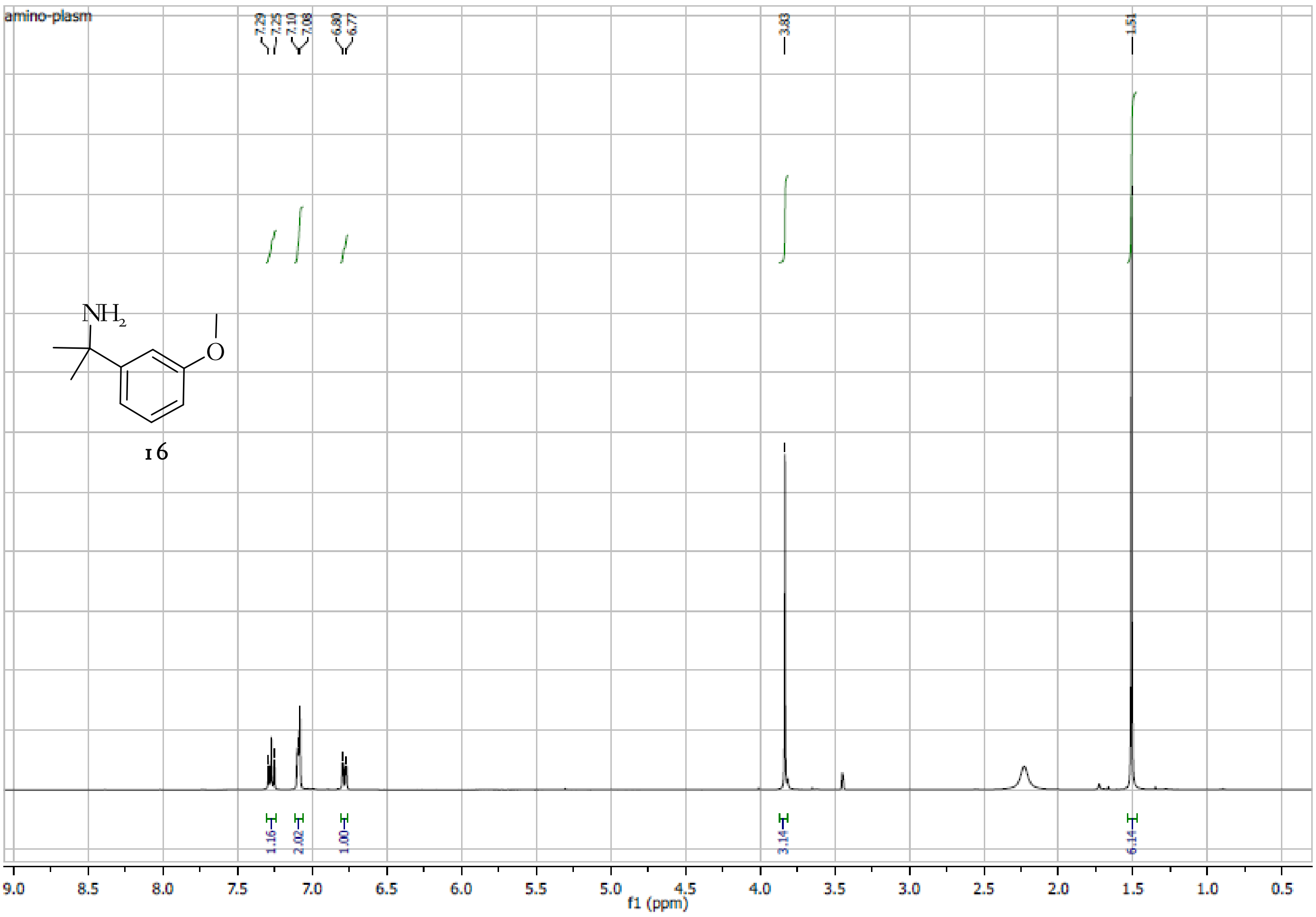

Figure S15. ^{1}H-NMR spectrum of **16**

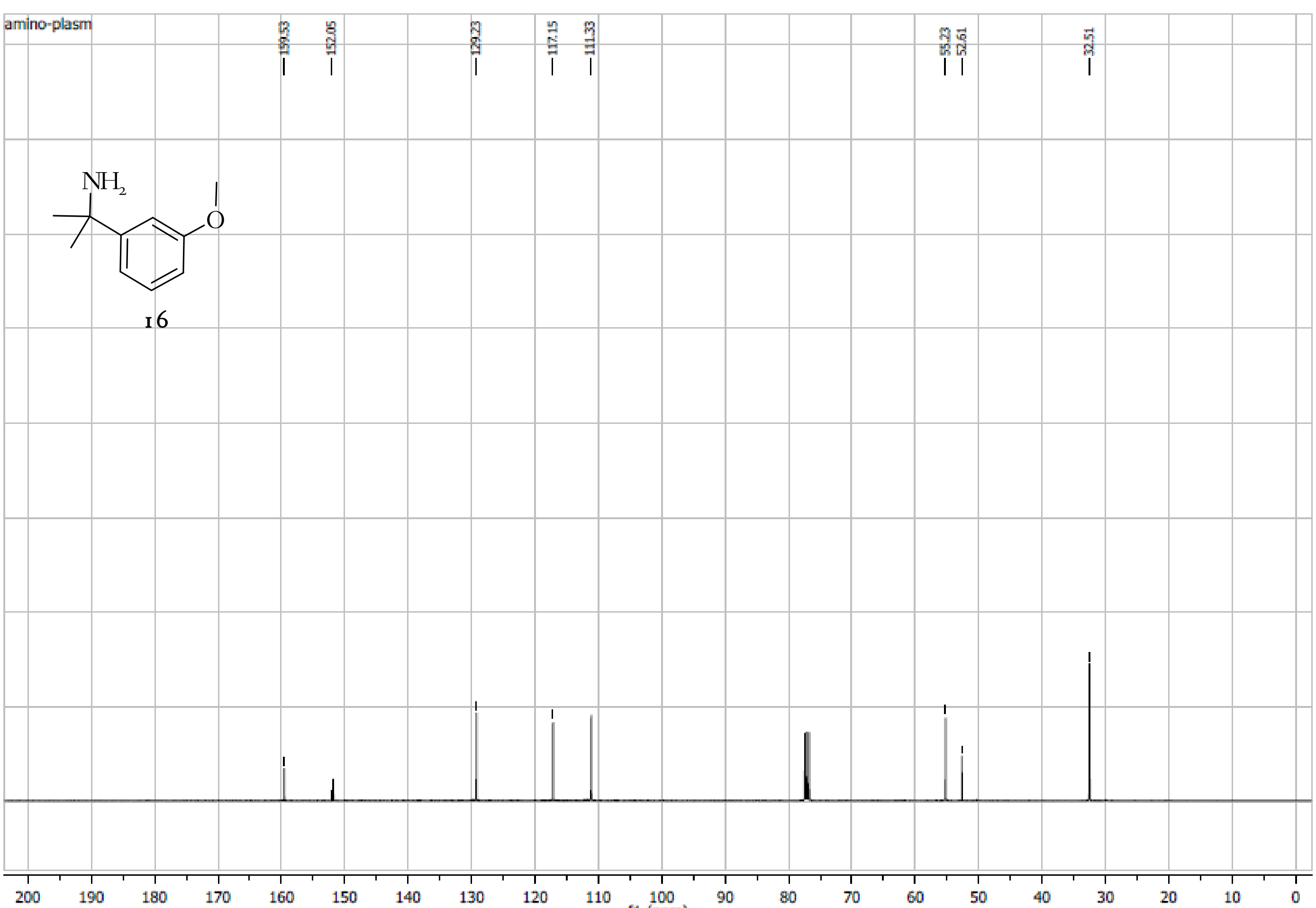

Figure S16. ^{13}C-NMR spectrum of **16**

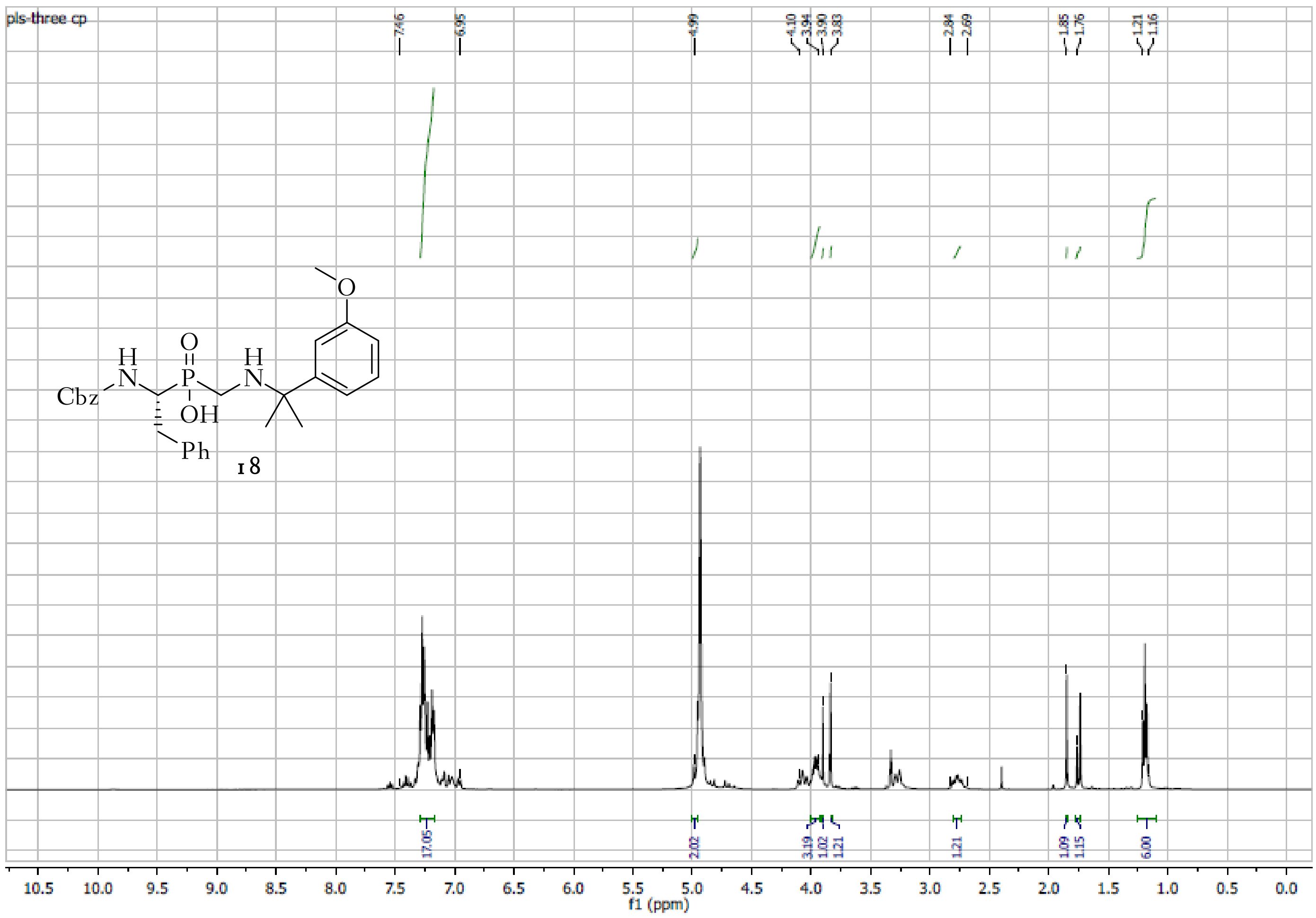

Figure S17. ^{1}H-NMR spectrum of **18**

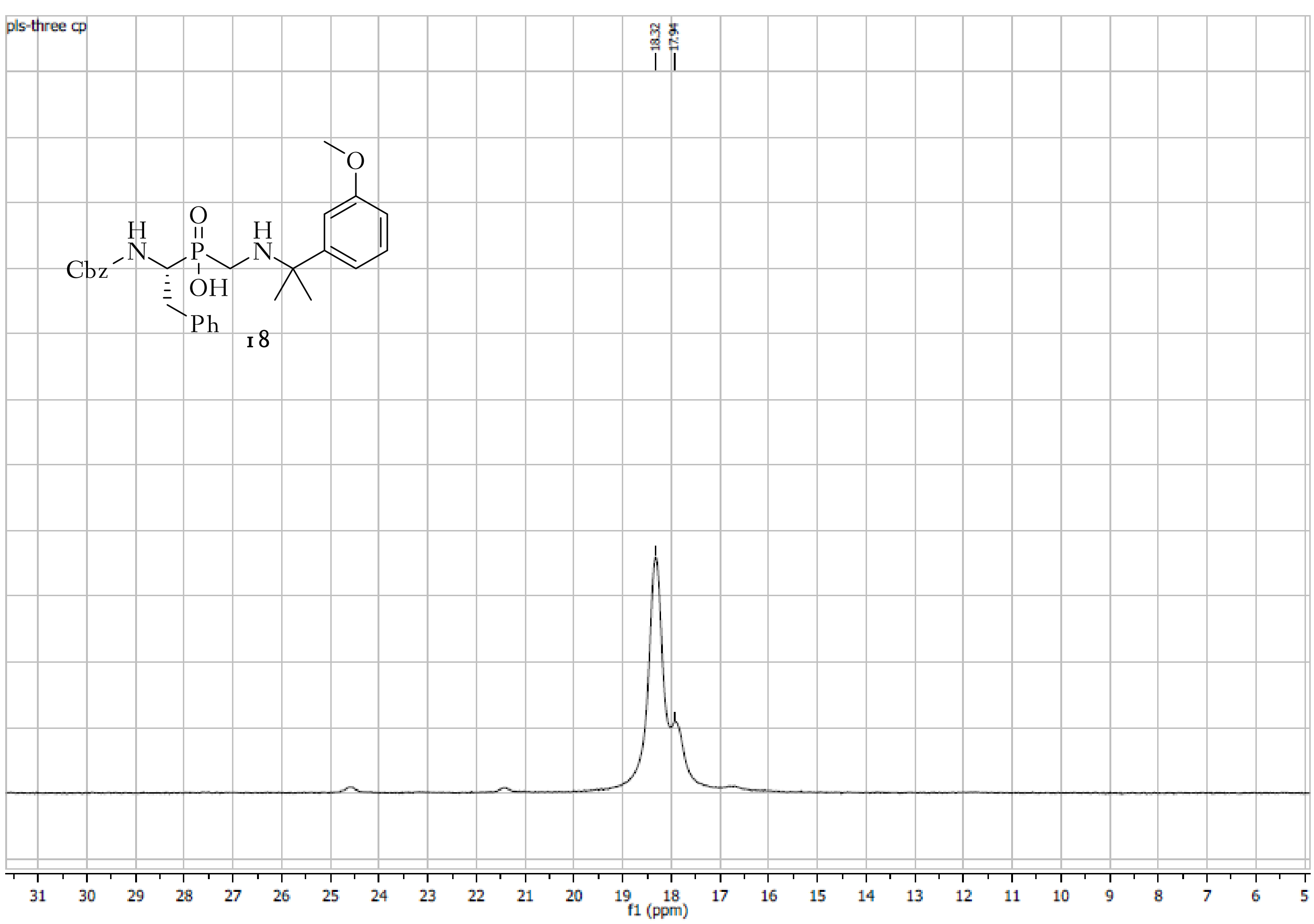

Figure S18. ^{31}P-NMR spectrum of **18**

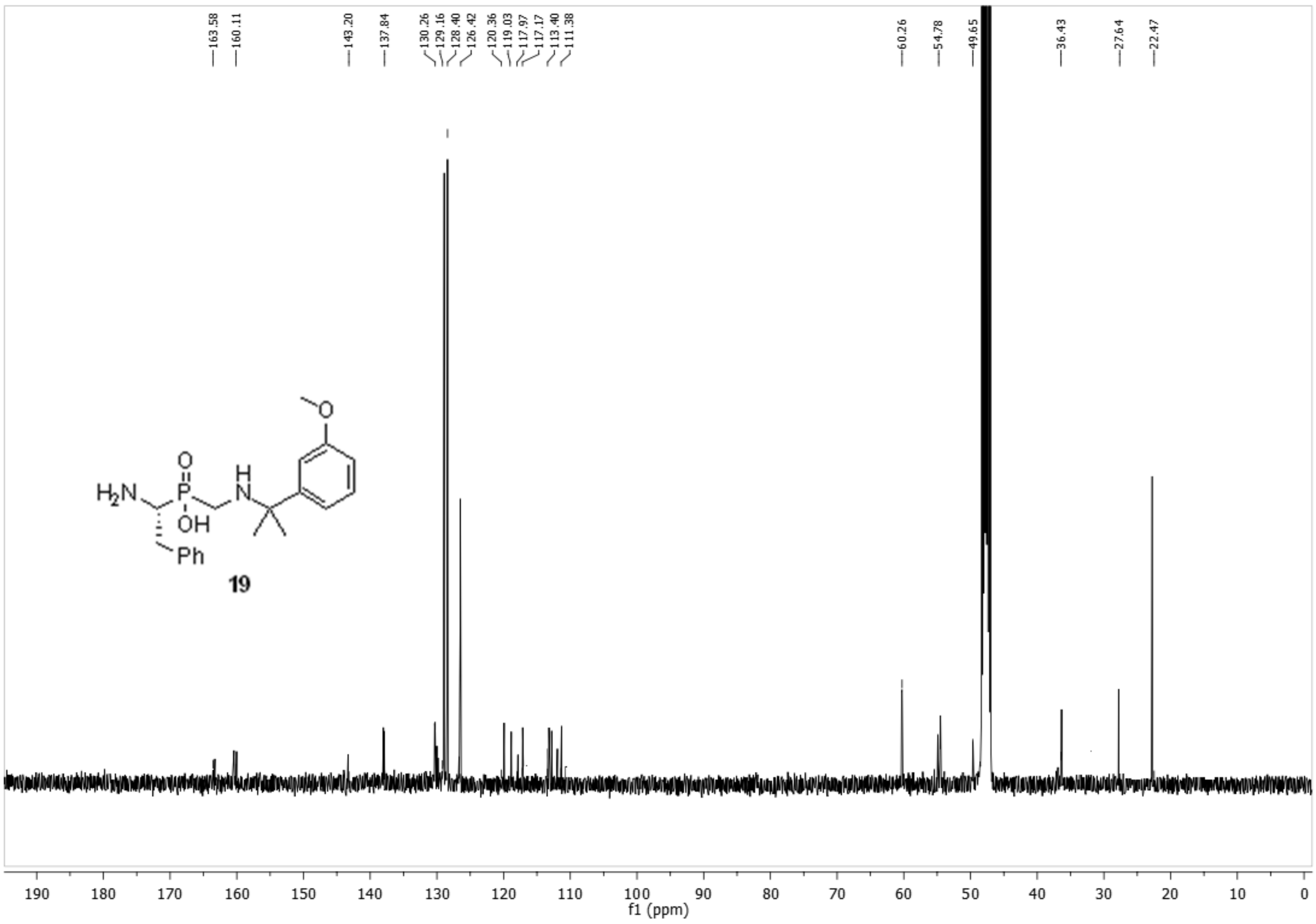

Figure S19. ^{13}C-NMR spectrum of **19**

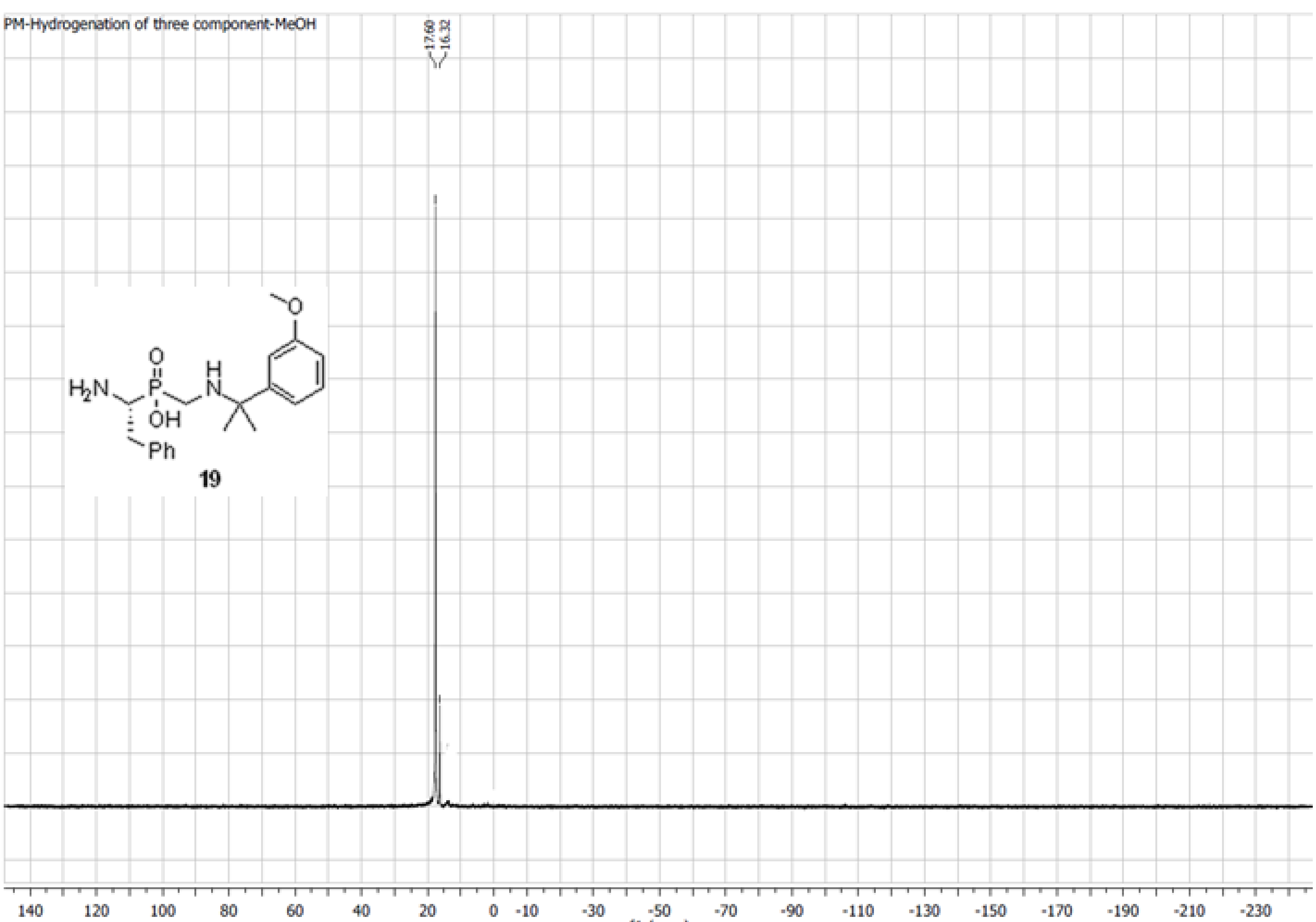

Figure S20. ^{31}P-NMR spectrum of **19**

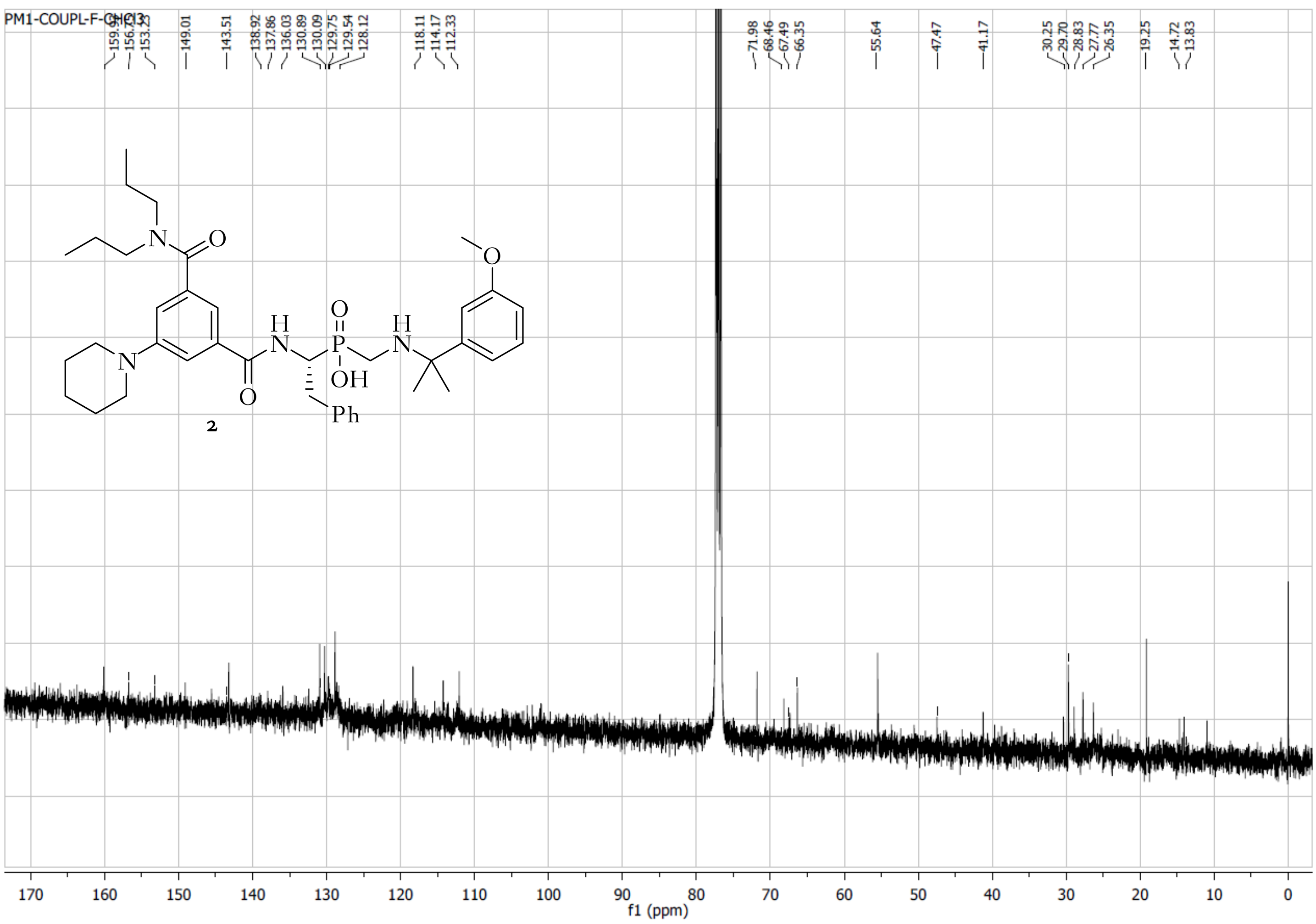

Figure S21. ^{13}C-NMR spectrum of **2**

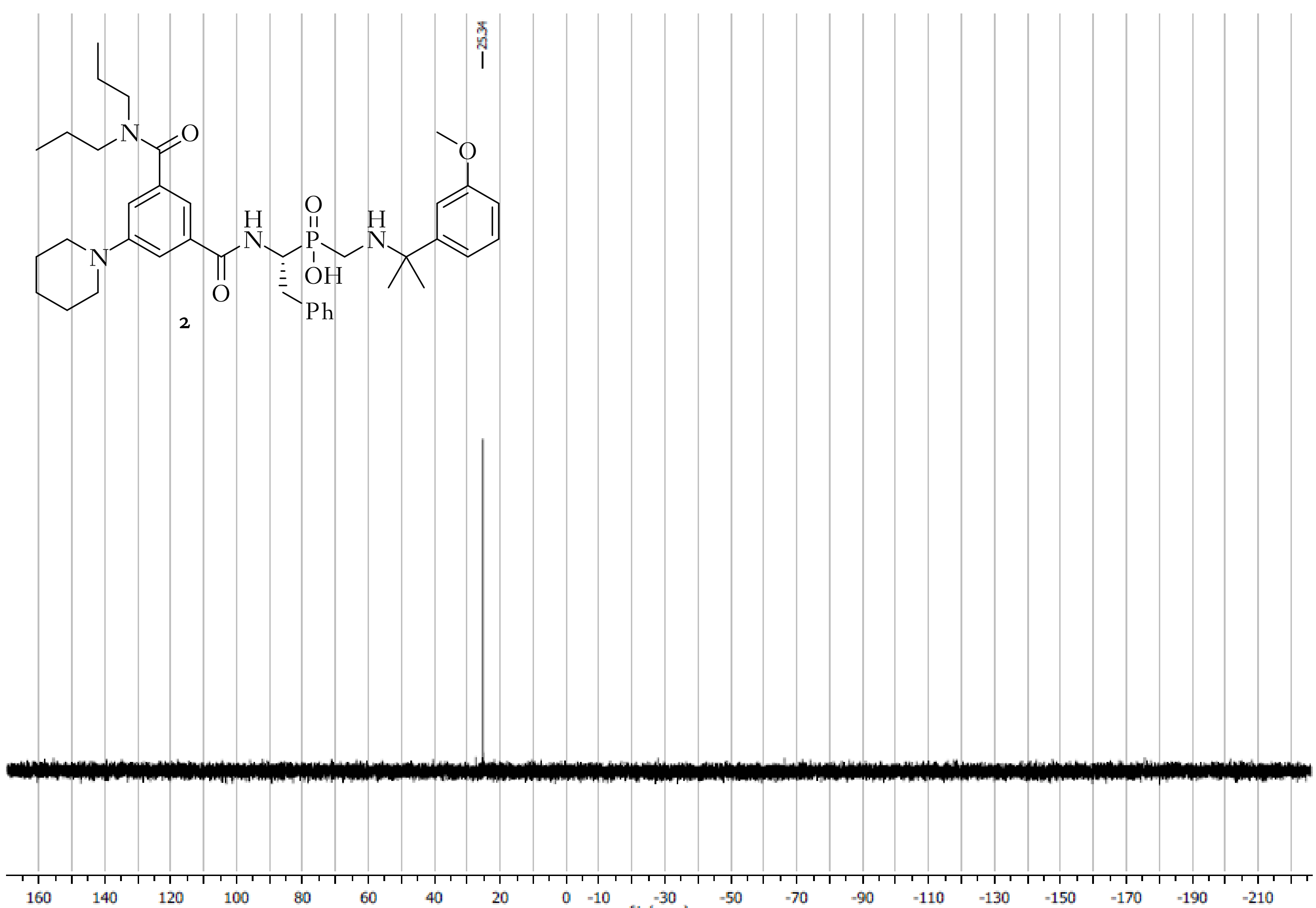

Figure S22. ^{31}P-NMR spectrum of **2**

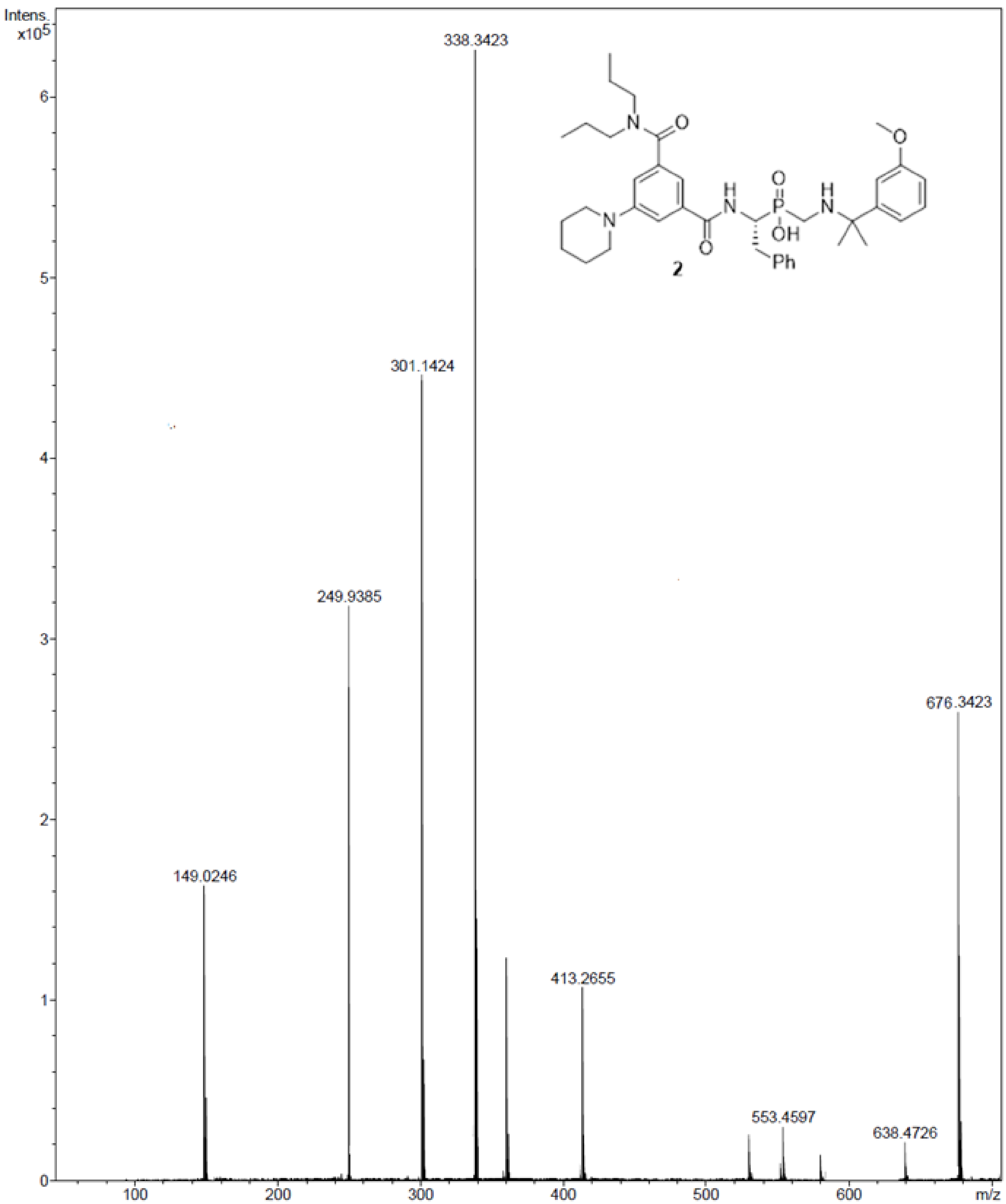

Figure S23. HRMS spectrum of **2**

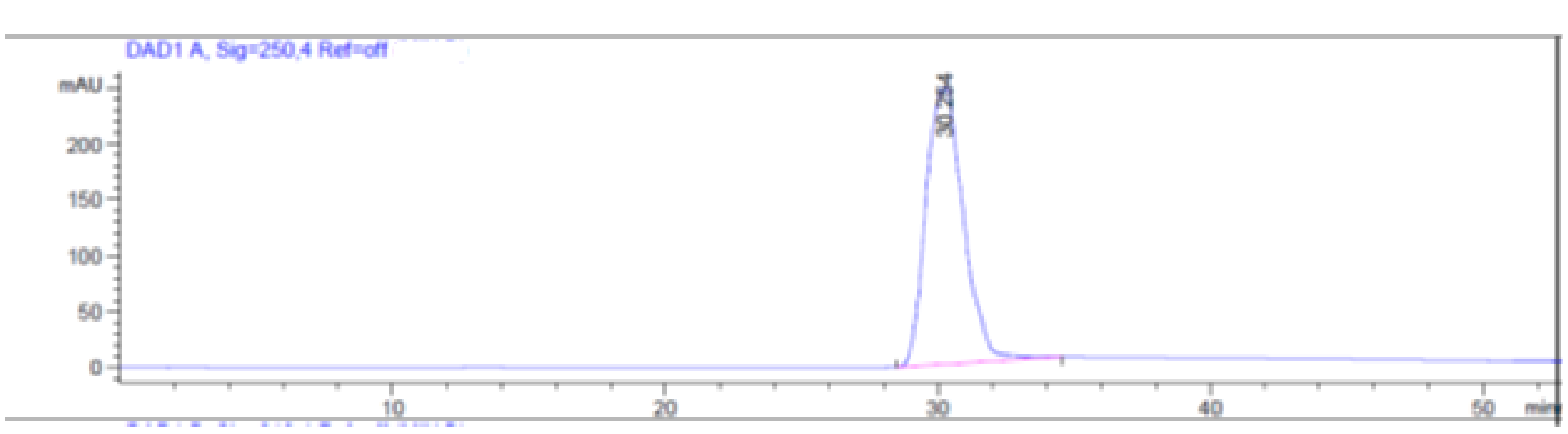

Figure S24. HPLC profile of **2**
